# Influence of winter temperature on nestling sex ratio in the cinereous vulture

**DOI:** 10.7717/peerj.21379

**Published:** 2026-06-08

**Authors:** Guillermo Gómez-López, Ana Sanz-Aguilar, Martina Carrete, Guillermo Blanco

**Affiliations:** 1 Department of Evolutionary Ecology, National Museum of Natural Sciences, CSIC, Madrid, Spain; 2 Animal Demography and Ecology Group, Institut Mediterrani d’Estudis Avançats, CSIC, Esporles, Mallorca, Spain; 3 Applied Zoology and Conservation Group, Universitat de les Illes Balears, Palma, Mallorca, Spain; 4 Department of Physical, Chemical and Natural Systems, Universidad Pablo de Olavide, Sevilla, Spain

**Keywords:** Offspring sex ratio, Nestling sex, *Aegypius monachus*, Monomorphism, Climatic conditions, Demography

## Abstract

For decades, variation in offspring sex ratios in birds has been a key topic in animal demography and population dynamics. In raptors, different factors such as food availability and breeding phenology may significantly influence sex ratios, while the impact of climatic conditions remains poorly documented. Here, we explored individual and environmental factors potentially affecting the secondary sex ratio of the long-lived, single-egg layer cinereous vulture *Aegypius monachus*. Using data collected over a 20-year period (2004–2023) in two breeding areas of central Spain, we assessed the effects of food availability, breeding phenology, climatic conditions, and spatiotemporal variability on offspring sex ratio (*n* = 186 nestlings). Offspring sex ratio was not biased either at the population, area or nucleus level, but deviations from a paired sex ratio were found in specific years and nuclei. Offspring sex ratio was not influenced by a food availability proxy (a two-level factor reflecting changes in livestock carcass availability derived from sanitary regulations implemented after the mad-cow crisis), and showed a tendency to be affected by breeding phenology, with more male offspring at the end of the breeding season in the high-altitude area. Moreover, temperature in the months prior to fertilization, but not after, affected nestling sex. Both cold and warm winters resulted in a predominance of male nestlings, while intermediate temperatures were associated with more females. Although the underlying mechanisms remain unclear, the temperature-associated sex ratio bias may be due to female sex manipulation or as an epiphenomenon associated with hormonal-mediated sex determination. These results emphasize the importance of studying how pre-fertilization climatic conditions might affect sex determination, and suggest that deviations in offspring sex ratios in long-lived species may be shaped by complex interactions between individual and environmental factors.

## Introduction

Offspring sex ratio, generally expressed as the proportion of males to females in a population, is a fundamental demographic trait that influences population dynamics, genetic diversity and evolutionary processes (Navara, 2018). In birds, this trait has attracted attention for decades (Mayr, 1939; Payevsky, 2021), but its underlying mechanisms and evolutionary drivers remain poorly understood (Navara, 2018). Fisher’s sex allocation theory postulates that natural selection favours an even offspring sex ratio (1:1) in species where the cost of raising male and female offspring is similar (Fisher, 1930). This has been confirmed in studies across several avian groups (Clutton-Brock, 1986; Gowaty, 1993; Donald, 2007; Gómez-López et al., 2023a). However, in species where this cost is different, such as those with sexual size dimorphism, differential parental investment in male and female offspring may result in a biased sex ratio (Komdeur & Pen, 2002; Szász, Kiss & Rosivall, 2012; Navara, 2018). Raptors, in particular, exhibit reversed sexual dimorphism, where females are the largest sex (Andersson & Norberg, 1981) and thus the more costly to raise (Frumkin, 1989; Anderson et al., 1993; Riedstra, Dijkstra & Daan, 1998). In addition to differential costs, environmental factors before or during the breeding season may bias the offspring sex ratio of raptors towards the sex that maximizes parental fitness (Trivers & Willard, 1973). For instance, years with limited resources often lead to a higher production of males, the less costly sex to raise (Wiebe & Bortolotti, 1992; Dzus, Bortolotti & Gerrard, 1996; Arroyo, 2002). Similarly, climatic conditions before or during the breeding season can affect offspring sex ratio (Hasselquist & Kempenaers, 2002; Sasvári & Nishiumi, 2005; Väli, 2012). For example, female-biased sex ratios have been associated with more rainfall in the preceding breeding season in lesser spotted eagles *Aquila pomarina* (Väli, 2012) and with less snow cover during the egg-laying period in tawny owls *Strix aluco* (Sasvári & Nishiumi, 2005). Most of these biases are ultimately linked to food availability, which enables parents to invest in the more costly sex when food resources are abundant and thus reduce its mortality risk (Trivers & Willard, 1973). However, other climatic variables like temperature may play a crucial role in sex determination, particularly in reptiles, where specific temperature ranges during the embryonic development influence the production of male or female offspring (Valenzuela & Lance, 2004).

Although limited information exists for raptors, studies in a few species of the orders Galliformes, Anseriformes and Passeriformes indicate that higher temperatures may favour the production of females, whereas lower temperatures may determine a bias towards males, which are less prone to mortality in such conditions (Eiby, Wilmer & Booth, 2008; Yilmaz et al., 2011; DuRant et al., 2016; Wada et al., 2018). This asymmetry between sexes may be due to different physiological responses to temperature stress in male and female embryos (Eiby, Wilmer & Booth, 2008; DuRant et al., 2016), especially in species with pronounced sex differences in size (Blanco et al., 2003a). However, these studies were done in artificial incubating conditions without female control, so the applicability of these results is limited (Eiby, Wilmer & Booth, 2008; Yilmaz et al., 2011; DuRant et al., 2016). Nevertheless, environmental conditions can affect sex allocation not only indirectly through sex differential mortality, but also directly through manipulation by the breeding female before or during fertilization, when sex determination occurs (Hasselquist & Kempenaers, 2002). Although some bird species can adjust offspring sex ratio in response to environmental conditions, the underlying physiological mechanisms, such as asynchronous follicular development, sex chromosome selection during the first meiotic division, and sex-specific ovulation, resorption, fertilization or inhibition of zygote formation, remain largely unknown (Krackow, 1995; Pike & Petrie, 2003; Navara, 2018). Notably, steroid hormones play a crucial role in translating environmental cues perceived by the female into physiological processes affecting sex determination (Navara, 2018).

Breeding phenology may also influence offspring sex ratio as a consequence of environmental, population and individual conditions, and several studies have shown that biases are particularly pronounced at the beginning or end of the breeding season (Daan, Dijkstra & Weissing, 1996; Smallwood & Smallwood, 1998; Ristow & Wink, 2004; Tschumi et al., 2019). The sex to be mainly produced early in the breeding season is often the one that will obtain a greater benefit from an earlier maturation and reproduction (Daan, Dijkstra & Weissing, 1996; Smallwood & Smallwood, 1998). The complex interplay of these and other individual and environmental factors affecting offspring sex ratio creates challenges identifying the exact causes of biases in each species, population or breeding pair (Hasselquist & Kempenaers, 2002; West, Reece & Sheldon, 2002; Gómez-López et al., 2023b).

To our knowledge, very few studies have thoroughly examined the environmental and individual factors that potentially influence variation in offspring sex ratio in vultures (Gómez-López et al., 2023a; Gómez-López et al., 2023b). In this study, we explored factors potentially influencing the secondary sex ratio in the cinereous vulture *Aegypius monachus*, a long-lived species that lays a single egg per year. We assessed variation in offspring sex ratio across different breeding nuclei with variable climatic conditions, as well as the effects of food availability and breeding phenology. Due to limited research in this topic, we tested whether temperature and rainfall prior to fertilization—when sex determination occurs—and after fertilization—when a sex-biased egg or nestling mortality may occur—may affect offspring sex ratio. Although we do not *a priori* expect strong sex ratio biases in this slightly dimorphic species, males are somewhat smaller (Glutz von Blotzheim, Bauer & Bezzel, 1971) and, presumably, less costly to produce than females. Thus, we predict that more males may be produced (i) in years with lower food availability and (ii) harsher climatic conditions (*e.g.*, lower temperatures, higher rainfall or snow), and (iii) in later clutches.

## Materials and Methods

### Study species and area

The cinereous vulture is an avian scavenger that inhabits forested hills and mountains at approximately 300 to 1,500 m.a.s.l. in south-western Europe, the Middle East and central and eastern Asia (Birdlife International, 2021). It feeds mainly on carrion of medium and large mammals, especially rabbits in the Mediterranean region, and occasionally on reptiles, birds and garbage (Donázar, 1993; Blanco & Hornero-Méndez, 2023). Although usually solitary, individuals congregate at feeding, breeding and roosting sites (Van Overveld et al., 2020). The European population is largely resident despite some small-scale dispersal movements, and adults show high fidelity to natal colonies, especially males (García-Macía et al., 2023a; García-Macía et al., 2023b).

Like other vultures, cinereous vultures are slightly size-dimorphic (Cramp & Simmons, 1980; Donázar, 1993): females typically have longer wings (approx. 3%) and weigh more (approx. 7%) than males (Glutz von Blotzheim, Bauer & Bezzel, 1971). They nest in large, mature trees (usually oak or pine), forming monogamous pairs that lay a single egg between February and April. Incubation lasts 50–62 days, both parents share incubation and offspring rearing, and nestlings become independent approximately seven months after hatching (Hiraldo, 1977; Donázar, 1993). This species is globally listed as “Near threatened” (Birdlife International, 2021).

**Figure 1:**
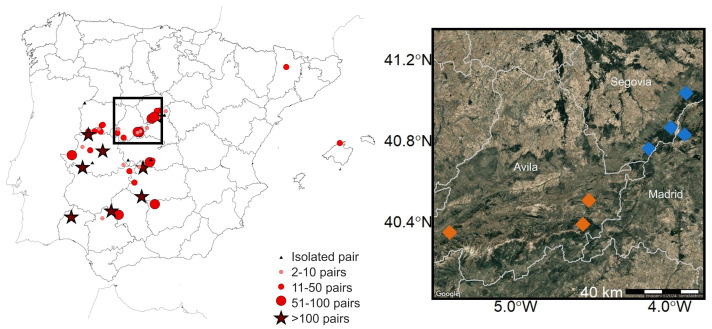
Distribution of cinereous vultures *Aegypius monachus* and location of the monitored breeding nuclei in central Spain. The breeding nuclei studied are located within the black square and the detailed map on the right. Stars and circles correspond to confirmed breeding nuclei of different sizes as recorded in the 2017 national census. Detailed map (on the right): nuclei from the high-altitude (blue) and low-altitude (orange) areas. Eastern Gredos nucleus is the top right orange point. Figure modified from Del Moral (2017). Map credit & data: Juan Oltra, ©2024 Google.

The Iberian Peninsula represents the main stronghold of this species in Europe, holding over 90% of the total population (Del Moral, 2017). We monitored two of its northernmost breeding areas in Spain, located in the provinces of Madrid, Segovia and Ávila (Fargallo, Blanco & Soto-Largo, 1998; Donázar et al., 2002). This region is home to ca. 15% of the Spanish breeding population (Del Moral, 2017) and is characterized by a continental Mediterranean climate with cold winters and hot summers, rugged orography and a mixture of natural vegetation and agricultural mosaics. The low-altitude area encompasses three breeding nuclei (Fig. 1) distributed in dry Mediterranean woodlands of maritime pines (*Pinus pinaster*), oaks (*Quercus rotundifolia*) and junipers (*Juniperus communis*) in the eastern and central sectors of Sierra de Gredos, Ávila (900–1,300 m.a.s.l.) (Chakarov & Blanco, 2021). This area falls within the supra-Mediterranean bioclimatic stage, characterized by an annual mean temperature of 8–13 °C and cold winters (−1 to −4 °C; Rivas-Martínez, 1987). In contrast, the high-altitude area includes four breeding nuclei (Fig. 1) situated in humid montane pinewoods of Scots pines (*Pinus sylvestris*) in Sierra de Guadarrama, between Madrid and Segovia (1,300–2,000 m.a.s.l.) (Chakarov & Blanco, 2021). This area falls within the supra- and the oro-Mediterranean stages, the latter characterized by an annual mean temperature of 4–8 °C and very cold winters (−4 to −7 °C; Rivas-Martínez, 1987). Although cinereous vultures are typically Mediterranean, in the past they sought more inaccessible montane locations as refuges from human threats such as poisoning, shooting and nest robbing (Hiraldo, 1977; Cano et al., 2016). Each breeding nucleus includes active nests within an approximate 8-km range and is isolated from other nest aggregations. Breeding nuclei were sampled over different periods (see Table S1).

### Fieldwork and sampling procedures

In this study, we took advantage of long-term monitoring programs of cinereous vultures in two of their northernmost breeding areas in Spain (at low and high altitude, see above), collected from 2004 to 2023. During part (February to May) of each breeding season, surveys were conducted to detect breeding pairs and assess their breeding success (*i.e.,* nestling reaching fledging age). All observations were made with telescopes at long distances (>500 m) to minimise disturbances. In June and July, we climbed accessible, successful nests with nestlings aged 40–70 days to mark them with metal and plastic bands with unique alphanumeric codes. A blood sample (1 ml) was also collected from the brachial vein of each nestling and preserved in absolute ethanol for molecular sexing (Fridolfsson & Ellegren, 1999). Since we did not know the sex of nestlings that died before sampling, our analyses reflected only the secondary sex ratio. The wing length of nestlings was measured and their approximate age (in days) was estimated from a regression formula based on nestlings of known age (age = 1.001*wing length + 15.242), as both variables are linearly correlated in vultures (Donázar & Ceballos, 1989; Elósegui, 1989). This age estimation aligns with growth curves of cinereous vulture nestlings from southern Spain (Hiraldo, 1977). The hatching date of each nestling was estimated by back-calculation from the age at the banding date and expressed as a Julian date (Julian day number 1 assigned to 1 January). Samples from the Comunidad de Madrid were provided by the regional government (J. Vielva).

### Ethics statement

Field studies were conducted in accordance with guidelines of the Ethic Committee of CSIC (CEBA-EBD-12-56) and the Spanish Royal Decree 1205/2005 on the protection of animals for experimentation and other scientific research, and under permits from the Spanish Bird Ringing Centre (Permit Number: 530115) and the regional governments of Castilla y León (Dirección General del Medio Natural, Servicio de Espacios Naturales) and Madrid. No interventions aside from blood sampling were carried out (anaesthesia was not given since the procedure is very quick and essentially painless, and the risk of using anaesthesia can be high in raptors). Animals were captured, processed and released in a few minutes.

### Environmental variables

We used two approaches to study the variables affecting the sex ratio of cinereous vulture nestlings in the ecologically-different low- and high-altitude areas. First, we evaluated the effect of food availability and individual hatching date on the probability of a nestling being a male (see a similar approach in Gómez-López et al., 2023a). As a surrogate of food availability, we used a two-level factor reflecting changes in livestock carcass availability derived from sanitary regulations implemented after the mad-cow crisis, namely: (i) the restrictive period (2002–2011), during the outbreak of the Bovine Spongiform Encephalopathy, when strict sanitary regulations (*i.e.,* CE 1774/2002) prohibiting the abandonment of livestock carcasses dropped their availability by approx. 80% (Donázar, Margalida & Campión, 2009); and (ii) the post-restrictive period (2012 onwards), when more flexible regulations were progressively applied (*i.e.,* CE 142/2011 and RD 1632/2011) and food availability for scavengers gradually increased (Blanco, 2014; Morales-Reyes et al., 2017; Almaraz et al., 2022). We did not sample nestlings before 2001 (prior to the mad-cow crisis), when livestock carcasses were left in the field and were widely available as it was a legal and common practice.

Second, to evaluate potential effects of climatic variables on the probability of a nestling being a male, we calculated the approximate fertilization date of each nestling, assuming a standard period of 15 days before the laying date (Bertran, Macià & Margalida, 2016), which was estimated to be 55 days before the hatching date (50–62 days; Hiraldo, 1977). Once fertilization date was obtained, we evaluated several climatic variables for each nestling over periods from one to four months before fertilization, alone and grouped (*i.e.,* 0–30, 0–60, 0–90, 0–120, 30–60, 30–90, 30–120, 60–90, 60–120 and 90–120 days before the fertilization date), and after fertilization (*i.e.,* from fertilization to hatching date, and from hatching to banding date). We explored different combinations since we do not know the exact period in which environmental conditions could be affecting breeding females (*i.e.,* during ovulation or fertilization) or their offspring (*i.e.,* egg or nestling stage). For each period, we ensured that we had at least 15 days of climatic data. As climatic variables, we used different descriptors for temperature—mean minimum, maximum and average temperatures (°C), as well as their standard deviation and range—because of their potential influence on sex determination (Valenzuela & Lance, 2004), and one descriptor for rainfall—total rainfall (mm). More specifically, we started using the daily minimum and maximum temperatures to obtain the daily average temperature, and then we averaged each of the three variables for the desired period of each nestling.

Third, considering only the eastern Gredos nucleus—for which we had the longest data series and the largest sample size—, we tested the effects of the same climatic variables as well as for the entire winter season (between 1 November and 28 February) on the annual offspring sex ratio (*i.e.,* proportion of male to female nestlings sampled each year). All climatic data were obtained from the nearest meteorological station to each breeding nucleus (Spanish Meteorological Agency, AEMET).

### Statistical analyses

As we did not have an exhaustive sampling of all active nests and sample sizes were small in some cases, we used Monte Carlo simulations to evaluate whether sex ratios were significantly biased across the two breeding areas, seven breeding nuclei and 15 years of monitoring (Table S1). As previously described in Gómez-López et al., 2023b: for each breeding area, nucleus and year, we ran 1,000 simulations by randomly selecting a number of nestlings equal to its sample size from a theoretical set of 3,000 nestlings (arbitrary but sufficiently large number) with a paired sex ratio (1:1) and calculating the resulting sex ratio. Significance tests were generated by counting the number of randomized cases that resulted in a value greater/lower than the observed sex ratio of the area, nucleus or year and then dividing by 1,000 (*i.e.,* the total number of randomizations; Serrano, Carrete & Tella, 2008).

We ran univariate Generalized Linear Mixed Models (GLMM; binomial error distribution; logit link function) to explore individual effects and trends, and multivariate models to detect potential interactions between factors or confounding effects. Initially, all nestlings were included (*n* = 186). However, missing data for some variables limited sample size in the global analysis, so we decided to run separate analyses with different data subsets to maximize sample sizes for each tested variable (note that sample sizes varied depending on the factor(s) tested). The variance inflation factor (VIF) was utilised to assess collinearity between variables. All models included year as a random factor. Simulation-based power analyses (100 iterations) were conducted to evaluate the ability of each model to detect effects when sample sizes were small. Due to unmarked breeding adults and frequent nest collapses and rebuilding in nearby areas, we could not use identifiers (*e.g.*, breeding pair, nest or territory) to control for further pseudoreplication.

Model selection followed the Akaike Information Criterion corrected for small sample sizes (AICc; Burnham & Anderson, 2002). For each model set—comprising the null model but excluding any models that failed to converge—we computed ΔAICc (the difference between the AICc of a given model and that of the top-ranked model) as well as the Akaike weight (*w*) for each candidate model. All models within a set were fitted to the same dataset to ensure valid AICc comparisons. Models with ΔAICc ≤ 2 were regarded as equally supported (Burnham & Anderson, 2002). The strength of support for a given effect was classified as none, weak, or strong depending on whether the 95% confidence interval of its estimate substantially overlapped zero, marginally overlapped zero, or excluded zero, respectively. Parameter estimates are reported on the logit scale. Model diagnostics were conducted using the DHARMa package (Hartig, 2022), and all analyses were carried out in R version 4.1.2 (RStudio Team, 2021).

## Results

**Figure 2:**
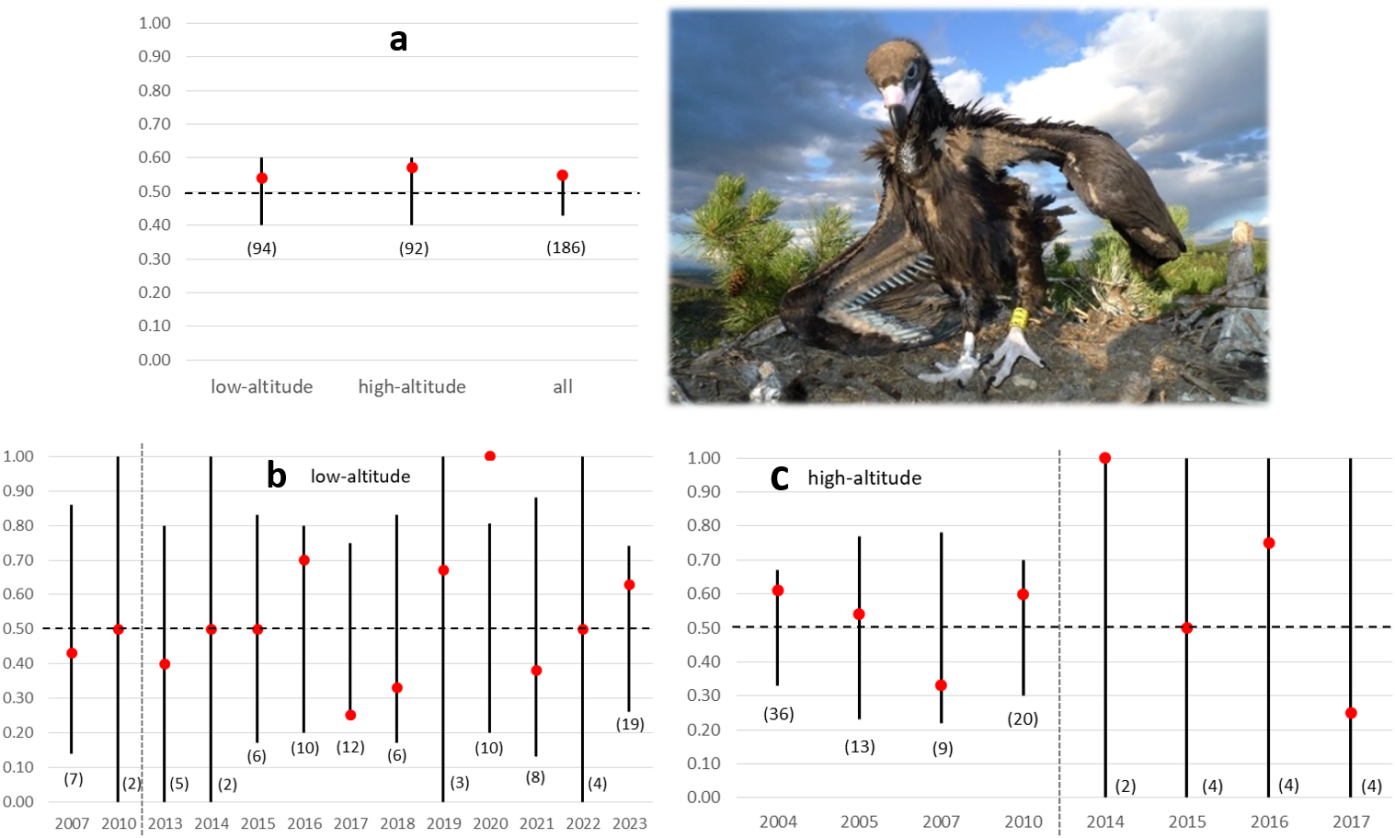
Observed and expected offspring sex ratios of cinereous vultures *Aegypius monachus* across sampled breeding areas and years, assuming a paired sex ratio (0.5) considering specific sample sizes. Sex ratios are defined as the proportion of males over total sexed nestlings. Observed sex ratios are represented with red dots, while expected sex ratios are shown as a horizontal dashed line (0.5). There were two breeding areas considered: low-altitude area (B) and high-altitude area (C). Sample sizes are shown in brackets. For each breeding area or year, 1,000 Monte Carlo simulations were conducted, randomly picking a number of nestlings equal to its sample size from a theoretical set of 3,000 nestlings with a paired sex ratio (1:1). The vertical black lines represent the 95% confidence intervals obtained from the simulations, while the vertical dashed line separates the years of the mad-cow crisis (2002–2011), when the dumping of livestock carcasses in the field was forbidden, and the post-crisis years (2012 onwards), when food availability for scavengers gradually increased (see Materials and Methods for details). Photo credit: Armando González.

Offspring sex ratio showed a non-significant male bias, both overall and across each breeding area (*n* = 186; Figs. 2A, 2B,2C). Significant deviation from a paired sex ratio occurred in specific breeding nuclei in specific years: there was a significant male bias in 2020 in a nucleus from the low-altitude area (eastern Gredos; sex ratio = 1.00; 95% CI [0.20–0.80]; *n* = 10; Fig. 2B), while there were marginally significant female biases in 2017 in the same nucleus (sex ratio = 0.25; 95% CI [0.25–0.75]; *n* = 12; Fig. 2B) and in 2007 in a nucleus from the high-altitude area (sex ratio = 0.17; 95% CI [0.17–0.83]; *n* = 6; Fig. 2C) (Table S1).

We found no significant effects of food availability on the probability of a nestling being a male (Table 1). Models including hatching date and its interaction with breeding area were ranked as alternatives to the null model (ΔAICc < 2), though the 95% CI of the estimates of these variables overlapped zero (Table 1). However, there was a tendency to find more male offspring at the end of the breeding season (mid-late May) in the high-altitude area (Table S2). Simulation-based power analyses showed that, as expected given our low sample sizes, the probability of detecting effects on offspring sex ratio was very low across models (95% CI [0.0–3.6]%; 0 of 100 simulations). Therefore, non-significant results should be interpreted with caution.

**Table 1:** Models assessing the effects of food availability, breeding area and hatching date on the probability of a nestling cinereous vulture *Aegypius monachus* being a male. Food availability (estimated based on the restrictive/post-restrictive periods of the mad-cow crisis): mad-cows; breeding area: area. (A) Models with all sexed nestlings (*n*  = 186); (B) models including only sexed nestlings of known hatching date (*n*  = 167). Estimates, standard errors (SE), and 95% confidence intervals (CI) are shown for alternative models only (ΔAICc < 2). Year was included as a random term in all models. The null model was included in both sets of models. df, degrees of freedom; AICc, Akaike information criterion corrected for small sample sizes; ΔAICc, difference between the AICc of model *i* and that of the best model (*i.e.*, the model with the lowest AICc); w, Akaike weight.

Model selection				
**Model**	**df**	**AICc**		**ΔAICc**	**w**
(A) all nestlings				
Null	2	258.12		0.00	0.56
Area	3	260.15		2.02	0.21
mad-cows	3	260.16		2.03	0.20
mad-cows*area	5	264.17		6.04	0.03
(B) Nestlings of known hatching date				
Null	2	232.34		0.00	0.38
Hatching date	3	232.85		0.51	0.29
Hatching date*area	5	233.65		1.31	0.20
Area	3	234.41		2.07	0.13
**Variable**	**Estimate**			**SE**	**2.5% CI**	**97.5% CI**
(B)				
Hatching date	0.21			0.17	−0.12	0.54
Hatching date	−0.11			0.24			−0.59	0.36
Area (high-altitude)	0.01			0.41	−0.79	0.81
Hatching date*area (high-altitude)	0.65			0.36	−0.06	1.37

### Effects of climatic conditions prior to fertilization

**Figure 3:**
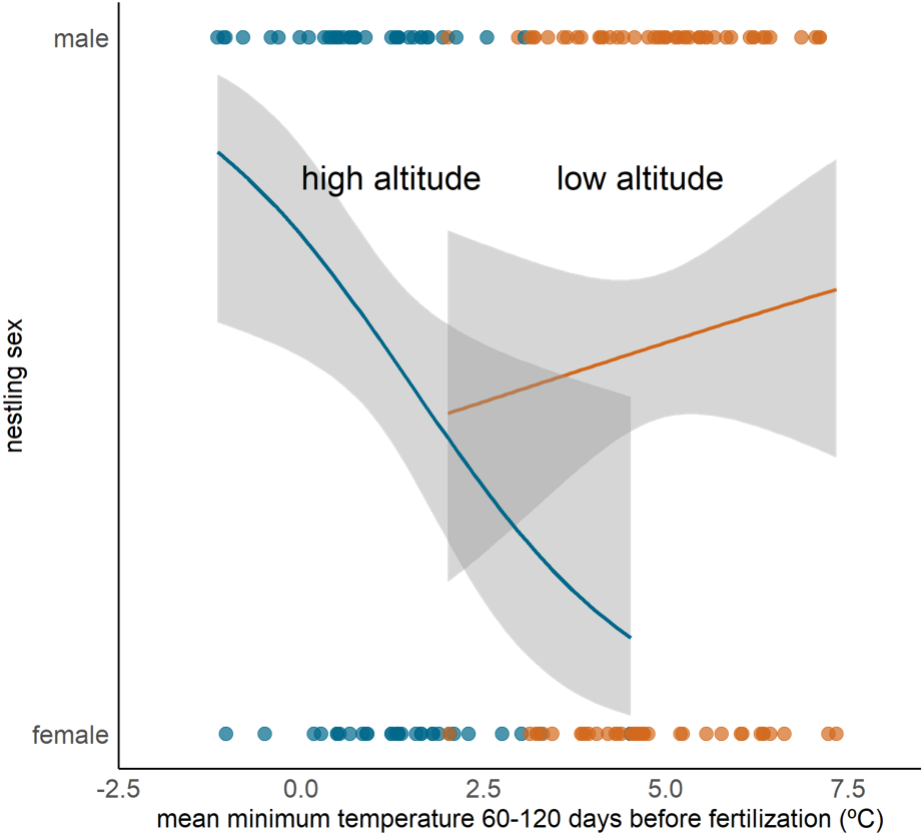
Relationship between the probability of a nestling cinereous vulture *Aegypius monachus* being a male and the mean minimum temperature (°C) during the 60–120 days before fertilization in two areas. In the high-altitude area (blue), the probability of a nestling being a male decreases as the mean minimum temperature increases; however, this relationship is not observed in the low-altitude area (orange). The grey area represents the 95% CI.

The mean minimum temperature during the 60–120 days before fertilization had a different effect on the probability of a nestling being a male in the high- and low-altitude areas (interaction mean minimum temperature during the 60–120 days before fertilization*area; Table 2; Fig. 3). Alternative models (ΔAICc < 2) included other temperature descriptors and total rainfall, but the 95% CI of their estimates overlapped zero (Table 2). When we analysed both areas separately, colder temperatures during the 60–120 days before fertilization increased the probability of a nestling being a male in the high-altitude area (mean for males = 0.8 °C; mean for females = 1.5 °C), while temperature had no effect in the low-altitude area (mean for males = 5.0 °C; mean for females = 4.8 °C) (Fig. 3; Table S3).

**Table 2:** Alternative models assessing the effects of the breeding area and climatic variables before fertilization on the probability of a nestling cinereous vulture *Aegypius monachus* being a male. Alternative models (ΔAICc < 2); breeding area: area; mean minimum temperature: min.mean; mean average temperature: avg.mean; total rainfall: rainfall; days before the fertilization date are shown between parentheses (see Materials and methods for details). (A) Models including nestlings with temperature data available (*n*  = 156); (B) models including nestlings with rainfall data available (*n*  = 139). Estimates, standard errors (SE), and 95% confidence intervals (CI) are shown. Year was included as a random term in all models. The null model was included in both sets of models. In bold, significant effects (*i.e.*, the 95% CI of the estimate does not overlap zero). df, degrees of freedom; AICc, Akaike information criterion corrected for small sample sizes; ΔAICc, difference between the AICc of model *i* and that of the best model (*i.e.*, the model with the lowest AICc); w, Akaike weight. All models run are shown in Table S4.

**Model selection**				
**Model**	**df**	**AICc**	**ΔAICc**	**w**
(A) Temperature				
min.mean (60–120)*area	5	215.94	0.00	0.08
Null	2	216.67	0.73	0.05
min.mean (90–120)	3	217.66	1.72	0.03
avg.mean (90–120)	3	217.89	1.95	0.03
min.mean (90–120)*area	5	217.90	1.96	0.03
(B) Rainfall				
Null	2	196.52	0.00	0.13
Rainfall (30–60)	3	196.53	0.01	0.13
Rainfall (30–90)	3	196.75	0.23	0.11
Rainfall (90–120)	3	197.93	1.41	0.06
Rainfall (30–60)*area	5	197.94	1.41	0.06
Rainfall (0–90)	3	198.09	1.57	0.06
Rainfall (60–90)	3	198.22	1.70	0.05
Rainfall (0–60)	3	198.32	1.79	0.05
Area	3	198.44	1.92	0.05
Rainfall (60–120)	3	198.50	1.97	0.05
**Variable**	**Estimate**		**SE**	**2.5% CI**	**97.5% CI**
(A)				
min.mean (60–120)	0.16		0.20	−0.23	0.55
Area (high-altitude)	1.53		1.11	−0.64	3.70
min.mean (60–120)*area (high-altitude)	**−0.77**		**0.33**	**−1.41**	**−0.13**
min.mean (90–120)	−0.08		0.08		−0.23	0.07
avg.mean (90–120)	−0.06		0.07		−0.20	0.07
min.mean (90–120)	0.03		0.13	−0.22	0.28
Area (high-altitude)	1.10		0.99	−0.84	3.04
min.mean (90–120)*area (high-altitude)	−0.37		0.20		−0.76	0.03
(B)				
Rainfall (30–60)	0.25		0.17	−0.09	0.59
Rainfall (30–90)	0.24		0.17	−0.10	0.58
Rainfall (90–120)	−0.15		0.18		−0.49	0.20
Rainfall (30–60)	−0.17		0.69		−0.94	0.61
Area (high-altitude)	−0.16		0.47		−1.07	0.75
Rainfall (30–60)*area (high-altitude)	0.76		0.49	−0.19	1.72
Rainfall (0–90)	0.13		0.18	−0.22	0.49
Rainfall (60–90)	0.11		0.18	−0.23	0.46
Rainfall (0–60)	0.10		0.19	−0.26	0.46
Area (high-altitude)	0.15		0.36	−0.55	0.86
Rainfall (60–120)	−0.06		0.18		−0.42	0.30

**Figure 4:**
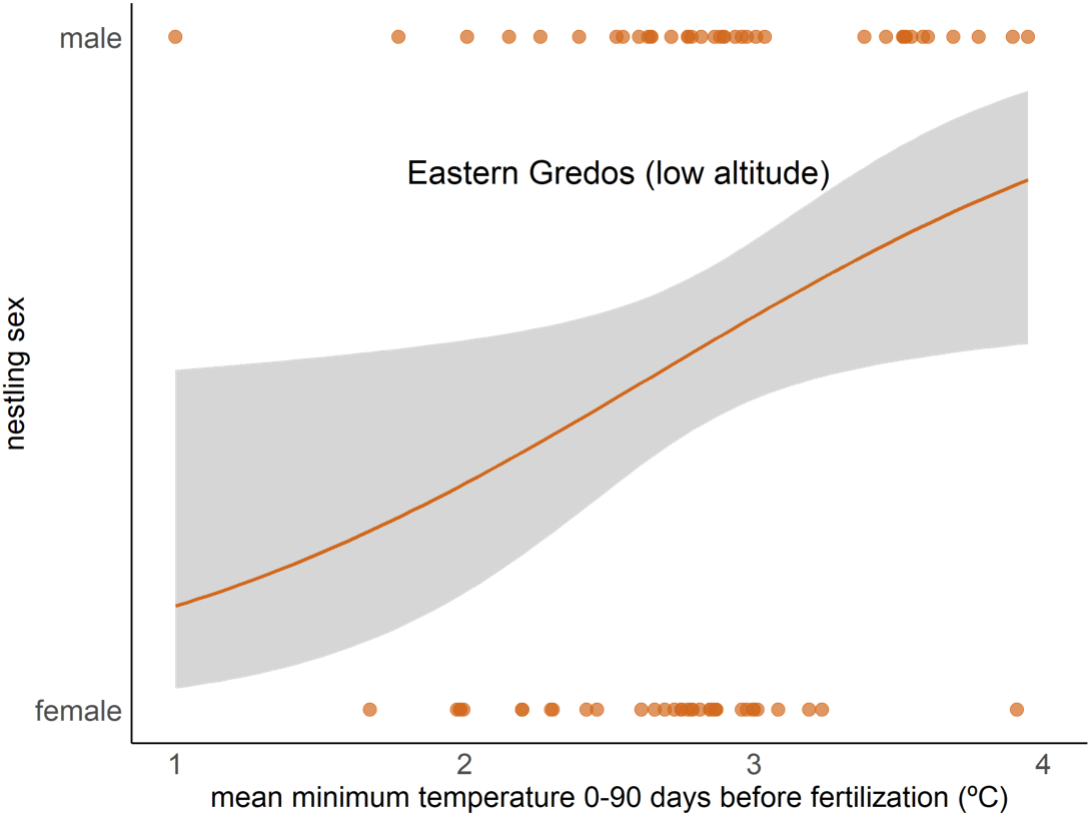
Relationship between the probability of a nestling cinereous vulture *Aegypius monachus* being a male in eastern Gredos and the mean minimum temperature (°C) during the 90 days before fertilization. The eastern Gredos nucleus is part of the low-altitude breeding area. The probability of a nestling being a male increases as mean minimum temperature increases. The grey area represents the 95% CI.

**Figure 5:**
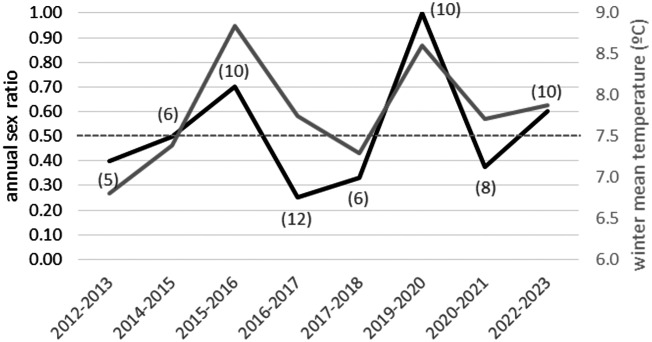
Annual secondary sex ratio of cinereous vultures *Aegypius monachus* in the eastern Gredos nucleus and winter mean temperature (°C). Sex ratio (solid black line) is defined as the proportion of males over the total nestlings sexed per year. Winter mean temperature is represented with a solid grey line. The eastern Gredos nucleus is part of the low-altitude breeding area. Only years with at least five nestlings sampled are considered in this graph. Sample sizes are shown in brackets. The horizontal dashed line marks a paired (1:1) sex ratio.

In the more detailed analysis using data from the eastern Gredos nucleus (low-altitude area), alternative models (ΔAICc < 2) obtained to assess the effect of temperature on the probability of a nestling being a male included mean minimum and average temperatures during the 90 days before fertilization in two separate models (Table 3). The 95% CI of the estimates of these variables did not overlap zero, suggesting that the probability of a nestling being a male increased with warmer temperatures during this period (Table 3; Fig. 4). Similarly, annual offspring sex ratio in this nucleus tended to be male-biased in warmer winters (Fig. 5; Table S5).

**Table 3:** Alternative models assessing the effects of temperature before fertilization on the probability of a nestling cinereous vulture *Aegypius monachus* being a male in eastern Gredos. Alternative models: ΔAICc < 2; mean minimum temperature: min.mean; mean maximum temperature: max.mean; mean average temperature: avg.mean; days before the fertilization date are shown between parentheses (see Materials and methods for details). *n* = 77 nestlings. Estimates, standard errors (SE), and 95% confidence intervals (CI) are shown. Year was included as a random term in all models. The null model was included in our set of models. In bold, significant effects (*i.e.*, the 95% CI of the estimate does not overlap zero). df, degrees of freedom; AICc, Akaike information criterion corrected for small sample sizes; ΔAICc, difference between the AICc of model *i* and that of the best model (*i.e.*, the model with the lowest AICc); w, Akaike weight. All models run are shown in Table S6.

**Model selection**				
**Model**	**df**	**AICc**	**ΔAICc**	**w**
min.mean (0–90)	3	106.23	0.00	0.09
avg.mean (0–90)	3	106.24	0.01	0.09
avg.mean (30–90)	3	106.82	0.60	0.07
min.mean (30–90)	3	107.18	0.95	0.06
max.mean (0–90)	3	107.31	1.08	0.05
max.mean (30–90)	3	107.33	1.10	0.05
avg.mean (60–90)	3	107.40	1.17	0.05
min.mean (60–90)	3	107.80	1.57	0.04
Null	2	108.19	1.96	0.04
**Variable**	**Estimate**	**SE**	**2.5% CI**	**97.5% CI**
**min.mean (0–90)**	**1.06**	**0.48**	**0**.**12**	**2.01**
**avg.mean (0–90)**	**0.79**	**0.34**	**0**.**12**	**1.46**
avg.mean (30–90)	0.62	0.32	0.00	1.25
min.mean (30–90)	0.66	0.36	−0.05	1.38
max.mean (0–90)	0.50	0.26	0.00	1.01
max.mean (30–90)	0.47	0.26	−0.03	0.98
avg.mean (60–90)	0.46	0.27	−0.06	0.99
min.mean (60–90)	0.42	0.27	−0.11	0.95

**Figure 6:**
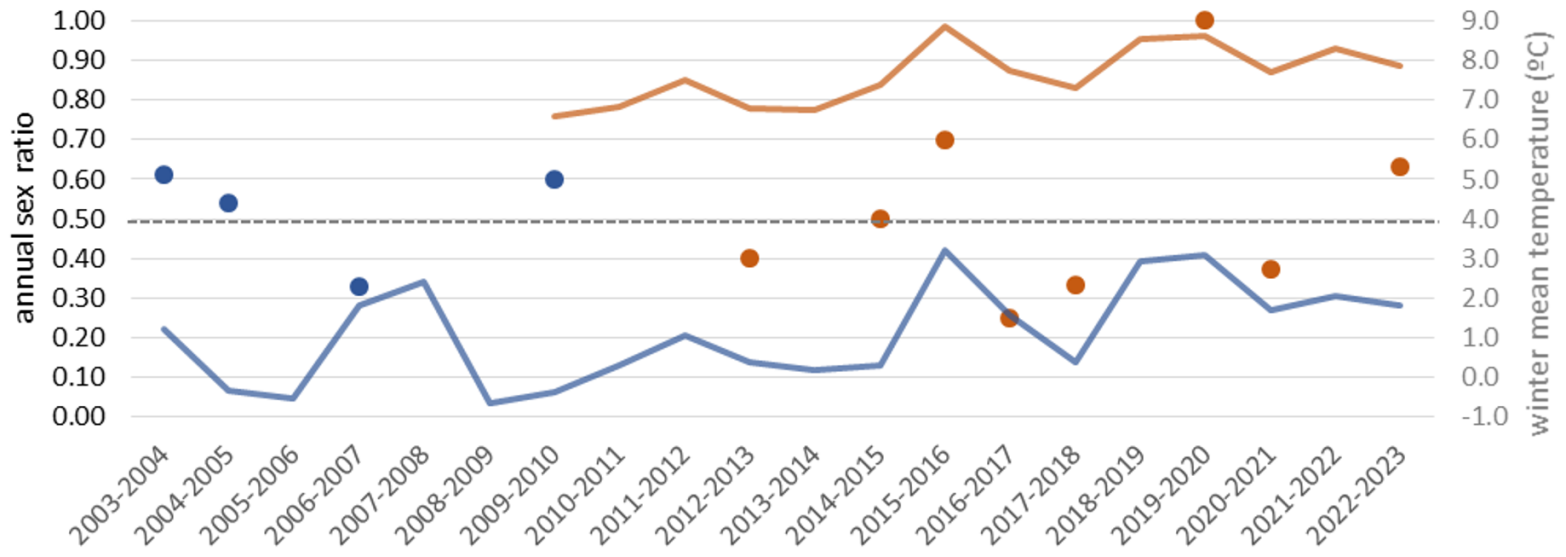
Relationship between the secondary sex ratio of cinereous vultures *Aegypius monachus* and winter mean temperature (°C) in two areas. Sex ratio (dots) is defined as the proportion of males over the total nestlings sexed per year. Winter mean temperature in each breeding area is represented with a solid line, either blue (high-altitude area) or orange (low-altitude area). Only years with at least five nestlings sampled are shown. The horizontal dashed line marks a paired (1:1) sex ratio. Note the substantial differences in winter temperatures between the two areas.

Comparing offspring sex ratios and winter mean temperatures in both the high- and low-altitude areas, we noted that the observed variations were not artefacts of the sampled years (Fig. 6). Notably, 2015–2016 and 2019–2020 (male-biased sex ratios) were among the three warmest winters in the time series for the low-altitude area, while 2006–2007 (female-biased sex ratio) was the second warmest winter in the first decade of the high-altitude area’s time series.

### Effects of climatic conditions after fertilization

**Figure 7:**
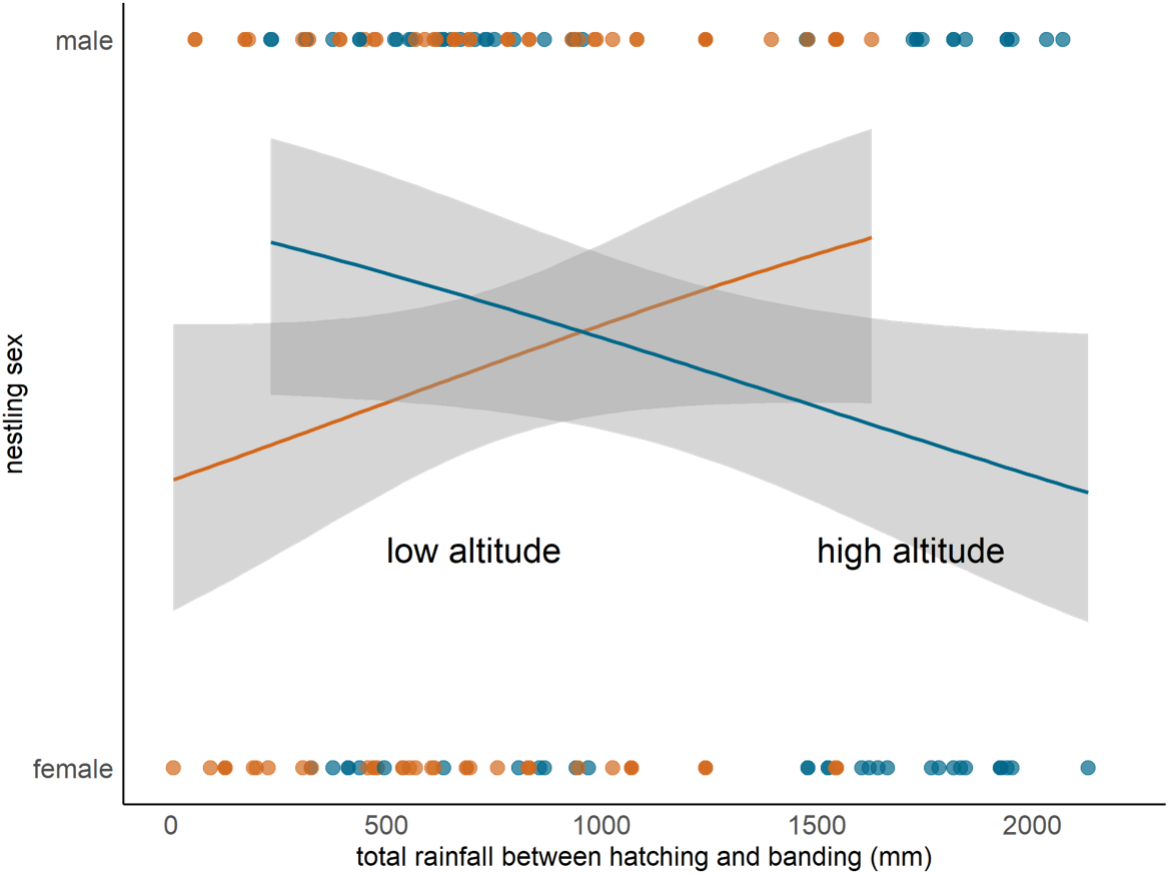
Relationship between the probability of a nestling cinereous vulture *Aegypius monachus* being a male and the total rainfall (mm) between hatching and banding in two areas. The sampled breeding areas are represented with blue (high-altitude area) and orange (low-altitude area). While the probability of a nestling being a male shows opposite trends between both areas, the effects are not significant. The grey area represents the 95% CI.

The total amount of rainfall between hatching and banding had a different effect on the probability of a nestling being a male in the high- and low-altitude areas (interaction total rainfall after hatching*area; Table 4; Fig. 7). Alternative models (ΔAICc < 2) also included temperature descriptors in separate models, but the 95% CI of their estimates overlapped zero (Table 4). When we analysed both areas separately, lower rainfall after hatching tended to increase the probability of a nestling being a male in the high-altitude area (mean for males = 1,036.9 mm; mean for females = 1,320.5 mm) while there was an opposite trend in the low-altitude area (mean for males = 845.5 mm; mean for females = 681.2 mm), but none of the effects were significant (Fig. 7; Table S7).

**Table 4:** Alternative models assessing the effects of the breeding area and climatic variables after fertilization on the probability of a nestling cinereous vulture *Aegypius monachus* being a male. Alternative models: ΔAICc < 2; breeding area: area; total rainfall: rainfall; range of the mean maximum temperature: max.range; range of the mean minimum temperature: min.range; two periods after the fertilization date were considered (see Materials and methods for details). *n* = 156 nestlings. Estimates, standard errors (SE), and 95% confidence intervals (CI) are shown. Year was included as a random term in all models. The null model was included in our set of models. In bold, significant effects (*i.e.*, the 95% CI of the estimate does not overlap zero). df, degrees of freedom; AICc, Akaike information criterion corrected for small sample sizes; ΔAICc, difference between the AICc of model *i* and that of the best model (*i.e.*, the model with the lowest AICc); w, Akaike weight. All models run are shown in Table S8.

**Model selection**				
**Model**	**df**	**AICc**	**ΔAICc**	**w**
Rainfall (hatch-band)*area	5	216.21	0.00	0.12
Null	2	216.67	0.46	0.10
max.range (hatch-band)	3	217.92	1.71	0.05
max.range (fert-hatch)	3	218.05	1.84	0.05
min.range (hatch-band)	3	218.20	1.99	0.04
**Variable**	**Estimate**		**SE**	**2.5% CI**	**97.5% CI**
Rainfall (hatch-band)	0.59		0.37	−0.12	1.31
Area (high altitude)	−0.08		0.47		−0.99	0.84
Rainfall (hatch-band)*area (high altitude)	**−1.06**		**0.44**	**−1.92**	** −0.19**
max.range (hatch-band)	−0.05		0.06		−0.17	0.06
max.range (fert-hatch)	0.05		0.06	−0.07	0.17
min.range (hatch-band)	−0.05		0.07		−0.19	0.08

In the more detailed analysis using data from the eastern Gredos nucleus (low-altitude area), total rainfall was retained in one of the alternative models (ΔAICc < 2) as a predictor of the probability of a nestling being a male. However, the 95% CI of its estimate overlapped zero, indicating no significant effects (Table S9).

## Discussion

Overall, the secondary sex ratio of nestling cinereous vultures did not deviate significantly from parity and remained consistently close to 1:1 over the years, as expected for a species with slight sexual dimorphism and balanced parental care (Glutz von Blotzheim, Bauer & Bezzel, 1971; Donázar, 1993). This finding, based on data spanning ca. 20 years in one of Europe’s key breeding areas for this species, is consistent with results observed in other cinereous vulture populations (Villegas et al., 2004) and other European vulture species (Bosé et al., 2007; López-López, Gil & Alcántara, 2011; Sanz-Aguilar et al., 2017; Davidovic et al., 2022; Gómez-López et al., 2023a; Gómez-López et al., 2023b). However, a closer examination revealed opposite patterns in three specific years: in 2020, offspring sex ratio was significantly male-biased (10 males, 0 females), while in 2007 and 2017 it was marginally female-biased (2007: five females, one male; 2017; nine females, three males). Notably, nestlings from 2020 and 2017 belonged to the eastern Gredos nucleus, located at one of the lowest altitudes of all sampled nuclei and, thus, with less cold winters. A similar non-significant male bias (13 males, seven females) was observed in one year in a south-western Spanish population of this species, probably due to poor environmental or parental conditions during breeding (Villegas et al., 2004). Such year-specific biases have also been documented in other raptor species, including the hen harrier *Circus cyaneus* (Picozzi, 1980), golden eagle *Aquila chrysaetos* (Edwards Jr et al., 1988), Harris’s hawk *Parabuteo unicinctus* (Bednarz & Hayden, 1991), northern goshawk *Accipiter gentilis* (Rutz, 2012) or eastern imperial eagle *Aquila heliaca* (Katzner et al., 2014). While these biases are typically considered exceptional events not linked to clear factors such as food availability or weather conditions (Katzner et al., 2014), their recurrence could suggest that they may not be purely stochastic, due to sample size limitations, but rather driven by complex interactions among factors.

The secondary sex ratio in our study population remained paired (1:1), regardless of variations in factors such as food availability (prediction (i) or hatching date (prediction (iii) but consistent with findings in other European vultures (Gómez-López et al., 2023a; Gómez-López et al., 2023b). However, in the high-altitude area, we found a tendency for more males to be born later in the breeding season, a pattern also seen in other raptors (Zijlstra, Daan & Bruinenberg-Rinsma, 1992; Ristow & Wink, 2004; Mora, Delgado & Penteriani, 2010). As for food availability, measured here using two distinctive periods of the mad-cow crisis as a proxy, we did not detect any influence on offspring sex ratio, although this does not mean that food availability is irrelevant for sex determination in vultures. Instead, it should be noted that quantifying carrion availability from both livestock and wild mammals is extremely hard, especially given the large areas over which individuals forage (Gómez-López et al., 2023b).

Climatic conditions, specifically pre-fertilization temperatures, apparently influenced offspring sex ratio in our cinereous vulture populations. In the high-altitude area, colder temperatures during the 60–120 days before fertilization were associated with a higher probability of raising males (mean for males = 0.8 °C; mean for females = 1.5 °C). Conversely, in the eastern Gredos nucleus (located in the low-altitude area), warmer temperatures during the 90 days before fertilization were linked to male biases (mean for males = 3.0 °C; mean for females = 2.7 °C). Notably, in the eastern Gredos nucleus, two warm winters with a male-biased sex ratio were followed by colder winters with a non-significant female-biased sex ratio. In a study with Eleonora’s falcon *Falco eleonorae*, nests which were more exposed to heat stress during the breeding season produced more males (Xirouchakis et al., 2022) while, in our study area, griffon vultures *Gyps fulvus* nesting in trees—potentially facing harsher climatic conditions—produced more males than those breeding on cliffs (Gómez-López et al., 2023a). Extreme temperatures during the breeding season can impact egg survival, and some vulture species avoid laying or incubating in extreme heat to minimise physiological stress on the eggs (Newton & Newton, 1996; Xirouchakis, 2010). In cinereous vultures, low temperatures during the incubation period (February–April) and high temperatures during the nestling period (May–July) may reduce breeding success (Hiraldo, 1977; Morán-López et al., 2006a). Indeed, despite their preference for high, steep slopes, cinereous vultures tend to avoid nesting sites with extreme summer and winter temperatures (Morán-López et al., 2006b). While the relationship is complex, our findings suggest that pre-fertilization temperatures may play a significant role in sex determination, supporting our prediction (ii) that harsher climatic conditions might be associated with a higher production of males—the potentially less costly sex—, especially after particularly harsh winters in the high-altitude area (oro-Mediterranean bioclimatic stage), characterized by colder temperatures and a greater number of rainy and snowy days per year. Meanwhile, more females might be produced more frequently when conditions are more benign. We could not find clear reasons for the opposite sex ratio pattern (higher production of males associated with warmer temperatures) in the low-altitude area, which is characterized by milder winters (supra-Mediterranean bioclimatic stage). Notably, we found no evidence that post-fertilization temperatures (*i.e.,* during egg and nestling development) affected sex ratio, which suggests that climatic effects on sex determination may be more relevant during the earliest stages of the vultures’ breeding cycle.

Previous studies have also shown that the breeding success of griffon vultures can be negatively correlated with the number of rainy days in spring (Xirouchakis, 2010) and with mean annual rainfall (Aresu et al., 2022), probably because rainfall restricts flight activity and hence foraging efficiency, especially in winter (Hiraldo, 1977). Similarly, Donázar et al. (2002) reported that rainy conditions during the nestling period (May–July) were associated with lower breeding success in cinereous vultures. In contrast with these effects on reproductive performance, our results indicate that rainfall did not have a clear or consistent influence on offspring sex ratio. Nevertheless, we observed opposing post-fertilization trends between both breeding areas studied: in the low-altitude area, the probability of raising males increased with higher rainfall, whereas in the high-altitude area, it increased under lower rainfall conditions. These contrasting patterns suggest that rainfall may interact with local environmental conditions rather than exerting a uniform effect on sex ratio. Given the substantial differences between breeding areas in factors such as temperature regimes, altitude or rainfall, further targeted studies would be necessary to disentangle the mechanisms underlying these area-specific responses.

Overall, our results indicate that even though pre-fertilization temperatures may bias the sex ratio of nestling cinereous vultures, the patterns observed are complex and appear to depend on inter-annual variations in environmental conditions as well as on nucleus-specific traits such as altitude. Unfortunately, our data did not allow us to evaluate the physiological mechanisms involved in sex allocation in breeding females in response to particular environmental cues. In addition to sex manipulation, biased offspring sex ratios may also arise from differential mortality between sexes (Krackow, 1995; Dijkstra, Daan & Pen, 1998; Nager et al., 2000). Thus, if one sex is more susceptible to specific environmental conditions during the incubation or nestling period, a higher mortality of such sex may bias sex ratio (Krackow, 1995; Nager et al., 2000). However, our results do not seem to support this mechanism, as neither temperature nor rainfall after fertilization was associated with detectable shifts in sex ratio. This suggests that the sex ratio biases observed in our population are more likely established prior to fertilization rather than being driven by sex-specific mortality during early development. It should also be noted that, while several models including climatic predictors showed support and identified significant effects, the null model was consistently among the top-ranked models, which indicates that the overall evidence for strong effects is limited.

Most studies on parental sex ratio manipulation in raptors have focused on species exhibiting marked sexual dimorphism and multiple-nestling broods (Wiebe & Bortolotti, 1992; Dzus, Bortolotti & Gerrard, 1996; Korpimäki et al., 2000; Arroyo, 2002; Blanco et al., 2003b). However, female cinereous vultures lay only one egg per year, a trait that may enhance their potential control over offspring sex. This idea is supported by studies showing sex ratio biases in first-laid eggs of species with multiple-egg clutches (Bortolotti, 1986; Bednarz & Hayden, 1991; Blanco et al., 2002; Xirouchakis et al., 2022). Nevertheless, the main patterns observed in our study may not necessarily result from adaptive female sex manipulation, but they could rather reflect an epiphenomenon associated with hormone-mediated sex determination (Blanco et al., 2002). Under this scenario, an increased production of one sex following cold or warm periods would not represent an adaptive response per se. Instead, some temperature fluctuations might trigger hormonal changes in breeding females before, during or shortly after fertilization, ultimately biasing offspring sex ratios—for example, through the differential deposition of some steroid hormones in the eggs (Groothuis & Schwabl, 2008). Additionally, exploring the influence of temperature on offspring sex ratios in other avian species would be valuable, since few field studies have explicitly considered this factor. For instance, megapodes (family *Megapodidae*) are the only known birds that do not incubate their eggs directly but rely on the heat produced within large mounds of soil and leaf litter, where the eggs are laid (Jones, Dekker & Roselaar, 1995). Males can actively regulate nest temperature by adding or removing material from these mounds, thereby influencing offspring sex ratios through temperature-dependent sex-biased embryo mortality (Göth & Booth, 2005; Eiby, Wilmer & Booth, 2008), a process extensively documented in reptiles (Valenzuela & Lance, 2004).

Several limitations should be acknowledged in our study. Firstly, the number of nestlings sampled varied considerably among years (three to 36) and breeding nuclei (40 to 94). Some of the explanatory variables considered (*e.g.*, hatching date, temperature, rainfall) were incomplete and unavailable for all individuals, preventing a comprehensive assessment of their potential effects on sex ratio. Very small sample sizes in certain nuclei or years further limited statistical power to detect subtle effects. Moreover, low temperatures may conceal snowfalls, blizzards or frosts, which could be biologically relevant; however, detailed data on such events were generally unavailable. It would be very useful to conduct a closer monitoring of the vulture pairs during the breeding season to improve biological data on fertilization, laying, hatching and nestling survival, and to confirm the present findings also for the primary sex ratio. Future studies might be able to improve our understanding of temperature effects on offspring sex ratio in cinereous vultures by assessing nestling physical condition following warmer *versus* colder winters, or under warmer climatic conditions in western Spain. For example, nestling body mass was reported to be lower in cinereous vultures from the high-altitude area compared to the low-altitude one, and in griffon vultures from tree nests compared to rock nests (Chakarov & Blanco, 2021), but the influence of temperature or rainfall in shaping these patterns was not explicitly tested. Finally, adverse environmental conditions may also be interacting with sex-specific genetic architecture and contribute to sex ratio variation through differential survival of male and female offspring: in birds, females are the heterogametic sex (ZW), so deleterious alleles located on the Z chromosome may be less effectively masked than in males and thus increase female vulnerability under stressful conditions (Liker & Székely, 2005). Unfortunately, our data did not allow us to evaluate this hypothesis.

## Conclusions

Overall, there are still substantial knowledge gaps regarding the biology of the cinereous vulture, particularly concerning physiology, reproduction and demography, which currently hindered our ability to draw robust conclusions on the proximate mechanisms driving sex determination and population sex ratio variations. Despite their preliminary nature, our results on offspring sex ratio are relevant in pointing out potential biases in a key demographic parameter for long-lived species and its relationship with temperature. These findings open up new research perspectives and emphasize the need to identify which environmental factors might influence sex determination. Furthermore, recent advances in molecular techniques, including genomics, transcriptomics and epigenetics, offer promising opportunities to study the genetic basis of offspring sex determination and sex ratio biases (Jax, Wink & Kraus, 2018; Mazzoleni et al., 2021; Ramos & Antunes, 2022). Integrating these approaches with ecological and individual-level data will be crucial for advancing our understanding of the complex mechanisms underlying sex allocation and its ecological and evolutionary implications.

##  Supplemental Information

10.7717/peerj.21379/supp-1 Supplemental Information 1 Supplementary tables

10.7717/peerj.21379/supp-2 Supplemental Information 2 Raw data (nestling, temperature and rainfall info)

10.7717/peerj.21379/supp-3 Supplemental Information 3 Codebook for raw data

## Additional Information and Declarations

### Data Availability

The following information was supplied regarding data availability: The data is available in the Supplemental Files. The data is available at Zenodo: - Gómez-López, G., Sanz-Aguilar, A., Carrete, M., & Blanco, G. (2026). Temperature sex ratio cinereous vulture [Data set]. Zenodo. https://doi.org/10.5281/zenodo.19469453.

## References

[ref-1] Almaraz P, Martínez F, Morales-Reyes Z, Sánchez-Zapata JA, Blanco G (2022). Long-term demographic dynamics of a keystone scavenger disrupted by human-induced shifts in food availability. Ecological Applications.

[ref-2] Anderson D, Reeve J, Gomez J, Weathers W, Hutson S, Cunningham H, Bird D (1993). Sexual size dimorphism and food requirements of nestling birds. Canadian Journal of Zoology.

[ref-3] Andersson M, Norberg RA (1981). Evolution of reversed sexual size dimorphism and role partitioning among predatory birds, with a size scaling of flight performance. Biological Journal of the Linnean Society.

[ref-4] Aresu M, Pennino MG, De Rosa D, Rotta A, Berlinguer F (2022). Modelling the effect of environmental variables on the reproductive success of Griffon Vulture (*Gyps fulvus*) in Sardinia, Italy. Ibis.

[ref-5] Arroyo BE (2002). Fledgling sex ratio variation and future reproduction probability in Montagu’s harrier, *Circus pygargus*. Behavioral Ecology and Sociobiology.

[ref-6] Bednarz JC, Hayden TJ (1991). Skewed brood sex ratio and sex-biased hatching sequence in Harris’s hawks. American Naturalist.

[ref-7] Bertran J, Macià FX, Margalida A (2016). How do colonial Eurasian Griffon Vultures prevent extra-pair mating?. PeerJ.

[ref-8] Birdlife International (2021). *Aegypius monachus*. Aegypius Monachus. The IUCN Red List of Threatened Species.

[ref-9] Blanco G (2014). Can livestock carrion availability influence diet of wintering red kites? Implications of sanitary policies in ecosystem services and conservation. Population Ecology.

[ref-10] Blanco G, Dávila JA, Septiem JA, Rodríguez R, Martínez F (2002). Sex-biased initial eggs favours sons in the slightly size-dimorphic Scops owl (Otus scops). Biological Journal of the Linnean Society.

[ref-11] Blanco G, Hornero-Méndez D (2023). Interspecific differences in plasma carotenoid profiles in nestlings of three sympatric vulture species. Current Zoology.

[ref-12] Blanco G, Martínez-Padilla J, Dávila JA, Serrano D, Viñuela J (2003a). First evidence of sex differences in the duration of avian embryonic period: consequences for sibling competition in sexually dimorphic birds. Behavioral Ecology.

[ref-13] Blanco G, Martínez-Padilla J, Serrano D, Dávila JA, Viñuela J (2003b). Mass provisioning to different-sex eggs within the laying sequence: consequences for adjustment of reproductive effort in a sexually dimorphic bird. Journal of Animal Ecology.

[ref-14] Bortolotti GR (1986). Influence of sibling competition on nestling sex ratios of sexually dimorphic birds. American Naturalist.

[ref-15] Bosé M, Le Gouar P, Arthur C, Lambourdière J, Choisy JP, Henriquet S, Lecuyer P, Richard M, Tessier C, Sarrazin F (2007). Does sex matter in reintroduction of griffon vultures *Gyps fulvus*?. Oryx.

[ref-16] Burnham KP, Anderson DR (2002). Model selection and multimodel inference: a practical information-theoretic approach.

[ref-17] Cano C, De la Bodega D, Ayerza P, Mínguez E (2016). El veneno en España.

[ref-18] Chakarov N, Blanco G (2021). Blood parasites in sympatric vultures: role of nesting habits and effects on body condition. International Journal of Environmental Research and Public Health.

[ref-19] Clutton-Brock TH (1986). Sex ratio variation in birds. Ibis.

[ref-20] Cramp S, Simmons KEL (1980). Handbook of the birds of Europe, the Middle East and North Africa. The birds of the Western Palearctic.

[ref-21] Daan S, Dijkstra C, Weissing FJ (1996). An evolutionary explanation for seasonal trends in avian sex ratios. Behavioral Ecology.

[ref-22] Davidovic S, Marinkovic S, Hribsek I, Patenkovic A, Stamenkovic-Radak M, Tanaskovic M (2022). Sex ratio and relatedness in the Griffon vulture (*Gyps fulvus*) population of Serbia. PeerJ.

[ref-23] Del Moral JC (2017). El buitre negro en España, población reproductora en 2017 y método de censo.

[ref-24] Dijkstra C, Daan S, Pen I (1998). Fledgling sex ratios in relation to brood size in size-dimorphic altricial birds. Behavioral Ecology.

[ref-25] Donald PF (2007). Adult sex ratios in wild bird populations. Ibis.

[ref-26] Donázar JA (1993). Los buitres ibéricos: biología y conservación.

[ref-27] Donázar JA, Blanco G, Hiraldo F, Soto-Largo E, Oria J (2002). Effects of forestry and other land-use practices on the conservation of cinereous vultures. Ecological Applications.

[ref-28] Donázar JA, Ceballos O (1989). Growth rates of nestling Egyptian Vultures Neophron percnopterus in relation to brood size, hatching order and environmental factors. Ardea.

[ref-29] Donázar JA, Margalida A, Campión D (2009). Vultures, feeding stations and sanitary legislation: a conflict and its consequences from the perspective of conservation biology (Munibe, 29).

[ref-30] DuRant SE, Hopkins WA, Carter AW, Kirkpatrick LT, Navara KJ, Hawley DM (2016). Incubation temperature causes skewed sex ratios in a precocial bird. Journal of Experimental Biology.

[ref-31] Dzus EH, Bortolotti GR, Gerrard JM (1996). Does sex-biased hatching order in bald eagles vary with food resources?. Ecoscience.

[ref-32] Edwards Jr TC, Collopy MW, Steenhof K, Kochert MN (1988). Sex ratios of fledgling golden eagles. The Auk.

[ref-33] Eiby YA, Wilmer JW, Booth DT (2008). Temperature-dependent sex-biased embryo mortality in a bird. Proceedings of the Royal Society B: Biological Sciences.

[ref-34] Elósegui I (1989). Vautour fauve (*Gyps fulvus*), Gypaete barbu (*Gypaetus barbatus*), Percnoptere d’Egypte (*Neophron percnopterus*): synthèse bibligraphique et recherches. Acta Biologica Montana.

[ref-35] Fargallo J, Blanco G, Soto-Largo E (1998). Forest management effects on nesting habitat selected by Eurasian black vultures (*Aegypius monachus*) in central Spain. Journal of Raptor Research.

[ref-36] Fisher RA (1930). The genetical theory of natural selection.

[ref-37] Fridolfsson A-K, Ellegren H (1999). A simple and universal method for molecular sexing of non-ratite birds. Journal of Avian Biology.

[ref-38] Frumkin R (1989). Egg quality, nestling development and dispersal in the Sparrowhawk (Accipiter nisus). Journal of Raptor Research.

[ref-39] García-Macía J, Álvarez E, Galán M, Iglesias-Lebrija JJ, Gálvez M, Plana G, Vallverdú N, Urios V (2023a). Age, season and sex influence juvenile dispersal in the Iberian cinereous vultures (*Aegypius monachus*). Journal of Ornithology.

[ref-40] García-Macía J, Álvarez E, Galán M, Iglesias-Lebrija JJ, Gálvez M, Plana G, Vallverdú N, Urios V (2023b). Home range variability and philopatry in Cinereous vultures (*Aegypius monachus*) breeding in Iberia. Avian Research.

[ref-41] Glutz von Blotzheim UN, Bauer KM, Bezzel E (1971). Handbuch der Vögel Mitteleuropas. Band 4. Falconiformes.

[ref-42] Gómez-López G, Martínez F, Sanz-Aguilar A, Carrete M, Blanco G (2023a). Nestling sex ratio is unaffected by individual and population traits in the griffon vulture. Current Zoology.

[ref-43] Gómez-López G, Sanz-Aguilar A, Carrete M, Arrondo E, Benítez JR, Ceballos O, Cortés-Avizanda A, De Pablo F, Donázar JA, Frías Ó, Gangoso L, García-Alfonso M, González JL, Grande JM, Serrano D, Tella JL, Blanco G (2023b). Insularity determines nestling sex ratio variation in Egyptian vulture populations. Ecology and Evolution.

[ref-44] Göth A, Booth DT (2005). Temperature-dependent sex ratio in a bird. Biology Letters.

[ref-45] Gowaty PA (1993). Differential dispersal, local resource competition, and sex ratio variation in birds. American Naturalist.

[ref-46] Groothuis TGG, Schwabl H (2008). Hormone-mediated maternal effects in birds: mechanisms matter but what do we know of them?. Philosophical Transactions of the Royal Society B: Biological Sciences.

[ref-47] Hartig F (2022). https://cran.r-project.org/web/packages/DHARMa/index.html.

[ref-48] Hasselquist D, Kempenaers B (2002). Parental care and adaptive brood sex ratio manipulation in birds. Philosophical Transactions of the Royal Society B: Biological Sciences.

[ref-49] Hiraldo F (1977). El buitre negro en la península ibérica. Población, biología general, uso de recursos e interacciones con otras aves. PhD thesis.

[ref-50] Jax E, Wink M, Kraus RHS (2018). Avian transcriptomics: opportunities and challenges. Journal of Ornithology.

[ref-51] Jones D, Dekker R, Roselaar C (1995). The Megapodes. Revue D’écologie (la Terre Et la Vie).

[ref-52] Katzner TE, Jackson DS, Ivy J, Bragin EA, Dewoody A (2014). Variation in offspring sex ratio of a long-lived sexually dimorphic raptor, the Eastern Imperial Eagle *Aquila heliaca*. Ibis.

[ref-53] Komdeur J, Pen I (2002). Adaptive sex allocation in birds: the complexities of linking theory and practice. Philosophical Transactions of the Royal Society B: Biological Sciences.

[ref-54] Korpimäki E, May CA, Parkin DT, Wetton JH, Wiehn J (2000). Environmental- and parental condition-related variation in sex ratio of kestrel broods. Journal of Avian Biology.

[ref-55] Krackow S (1995). Potential mechanisms for sex ratio adjustment in mammals and birds. Biological Reviews of the Cambridge Philosophical Society.

[ref-56] Liker A, Székely T (2005). Mortality costs of sexual selection and parental care in natural populations of birds. Evolution.

[ref-57] López-López P, Gil JA, Alcántara M (2011). Morphometrics and sex determination in the endangered bearded vulture (*Gypaetus barbatus*). Journal of Raptor Research.

[ref-58] Mayr E (1939). The sex ratio in wild birds. The American Naturalist.

[ref-59] Mazzoleni S, Němec P, Albrecht T, Lymberakis P, Kratochvíl L, Rovatsos M (2021). Long-term stability of sex chromosome gene content allows accurate qPCR-based molecular sexing across birds. Molecular Ecology Resources.

[ref-60] Mora O, Delgado M, Penteriani V (2010). Secondary sex ratio in eurasian eagle-owls: early-breeding females produce more daughters. Journal of Raptor Research.

[ref-61] Morales-Reyes Z, Pérez-García JM, Moleón M, Botella F, Carrete M, Donázar JA, Cortés-Avizanda A, Arrondo E, Moreno-Opo R, Jiménez J, Margalida A, Sánchez-Zapata JA (2017). Evaluation of the network of protection areas for the feeding of scavengers in Spain: from biodiversity conservation to greenhouse gas emission savings. Journal of Applied Ecology.

[ref-62] Morán-López R, Sánchez JM, Costillo E, Corbacho C, Villegas A (2006a). Spatial variation in anthropic and natural factors regulating the breeding success of the cinereous vulture (*Aegypius monachus*) in the SW Iberian Peninsula. Biological Conservation.

[ref-63] Morán-López R, Sánchez JM, Costillo E, Villegas A (2006b). Nest-site selection of endangered cinereous vulture (*Aegypius monachus*) populations affected by anthropogenic disturbance: present and future conservation implications. Animal Conservation.

[ref-64] Nager RG, Monaghan P, Houston DC, Genovart M (2000). Parental condition, brood sex ratio and differential young survival: an experimental study in gulls (*Larus fuscus*). Behavioral Ecology and Sociobiology.

[ref-65] Navara KJ (2018). Choosing sexes, mechanisms and adaptive patterns of sex allocation in vertebrates.

[ref-66] Newton SF, Newton AV (1996). Breeding biology and seasonal abundance of lappet-faced vultures *Torgos tracheliotus* in western Saudi Arabia. Ibis.

[ref-67] Payevsky VA (2021). Sex ratio and sex-specific survival in avian populations: a review. Biology Bulletin Reviews.

[ref-68] Picozzi N (1980). Food, growth, survival and sex ratio of nestling hen harriers circus *c. cyaneus* in Orkney. Ornis Scandinavica.

[ref-69] Pike TW, Petrie M (2003). Potential mechanisms of avian sex manipulation. Biological Reviews of the Cambridge Philosophical Society.

[ref-70] Ramos L, Antunes A (2022). Decoding sex: elucidating sex determination and how high-quality genome assemblies are untangling the evolutionary dynamics of sex chromosomes. Genomics.

[ref-71] Riedstra B, Dijkstra C, Daan S (1998). Daily energy expenditure of male and female Marsh Harrier nestlings. The Auk.

[ref-72] Ristow D, Wink M (2004). Seasonal variation in sex ratio of nestling Eleonora’s falcons. Journal of Raptor Research.

[ref-73] Rivas-Martínez S (1987). Memoria del mapa de series de vegetación de España. ICONA.

[ref-74] RStudio Team (2021). http://www.rstudio.com/.

[ref-75] Rutz C (2012). Brood sex ratio varies with diet composition in a generalist raptor. Biological Journal of the Linnean Society.

[ref-76] Sanz-Aguilar A, Cortés-Avizanda A, Serrano D, Blanco G, Ceballos O, Grande JM, Tella JL, Donázar JA (2017). Sex- and age-dependent patterns of survival and breeding success in a long-lived endangered avian scavenger. Scientific Reports.

[ref-77] Sasvári L, Nishiumi I (2005). Environmental conditions affect offspring sex-ratio variation and adult survival in tawny owls. The Condor.

[ref-78] Serrano D, Carrete M, Tella JL (2008). Describing dispersal under habitat constraints: a randomization approach in lesser kestrels. Basic and Applied Ecology.

[ref-79] Smallwood PD, Smallwood JA (1998). Seasonal shifts in sex ratios of fledgling american kestrels (Falco *sparverius paulus*): the early bird hypothesis. Evolutionary Ecology.

[ref-80] Szász E, Kiss D, Rosivall B (2012). Sex ratio adjustment in birds. Ornis Hungarica.

[ref-81] Trivers RL, Willard DE (1973). Natural selection of parental ability to vary the sex ratio of offspring. Science.

[ref-82] Tschumi M, Humbel J, Erbes J, Fattebert J, Fischer J, Fritz G, Geiger B, Harxen R, Van Hoos B, Hurst J, Jacobsen LB, Keil H, Kneule W, Michel VT, Michels H, Möbius L, Perrig M, Rößler P, Schneider D, Schuch S, Stroeken P, Naef-Daenzer B, Grüebler MU (2019). Parental sex allocation and sex-specific survival drive offspring sex ratio bias in little owls. Behavioral Ecology & Sociobiology.

[ref-83] Valenzuela N, Lance VA (2004). Temperature dependent sex determination in vertebrates.

[ref-84] Väli Ü (2012). Factors limiting reproductive performance and nestling sex ratio in the Lesser Spotted Eagle Aquila pomarina at the northern limit of its range: the impact of weather and prey abundance. Acta Ornithologica.

[ref-85] Van Overveld T, Blanco G, Moleón M, Margalida A, Sánchez-Zapata JA, De la Riva M, Donázar JA (2020). Integrating vulture social behavior into conservation practice. The Condor.

[ref-86] Villegas A, Sánchez-Guzmán JM, Costillo E, Corbacho C, Morán R (2004). Productivity and fledgling sex ratio in a Cinereous Vulture (*Aegypius monachus*) population in Spain. Journal of Raptor Research.

[ref-87] Wada H, Kriengwatana BP, Steury TD, MacDougall-Shackleton SA (2018). Incubation temperature influences sex ratio and offspring’s body composition in zebra finches (*Taeniopygia guttata*). Canadian Journal of Zoology.

[ref-88] West S, Reece S, Sheldon B (2002). Sex ratios. Heredity.

[ref-89] Wiebe KL, Bortolotti GR (1992). Facultative sex ratio manipulation in American kestrels. Behavioral Ecology and Sociobiology.

[ref-90] Xirouchakis SM (2010). Breeding biology and reproductive performance of Griffon Vultures *Gyps fulvus* on the island of Crete (Greece). Bird Study.

[ref-91] Xirouchakis SM, Botsidou P, Baxevani K, Andreou G, Tsaparis D (2022). Brood sex ratio variation in a colonial raptor, the Eleonora’s falcon, Falco eleonorae. Animal Behaviour.

[ref-92] Yilmaz A, Tepeli C, Garip M, Caǧlayan T (2011). The effects of incubation temperature on the sex of Japanese quail chicks. Poultry Science.

[ref-93] Zijlstra M, Daan S, Bruinenberg-Rinsma J (1992). Seasonal variation in the sex ratio of Marsh Harrier Circus aeruginosus Broods. Functional Ecology.

