# Supplementary material for: Influence of winter temperature on nestling sex ratio in the cinereous vulture
Source: PeerJ. 2026 Jun 8;14:e21379. doi: 10.7717/peerj.21379 (PMC13256116; doi:10.7717/peerj.21379)
Supplement: Supplemental Information 1 [file peerj-14-21379-s001.docx]

## Supplementary material

**Table S1**. Secondary sex ratios of nestling cinereous vultures *Aegypius monachus* in the sampled breeding nuclei and years. Sex ratios are expressed as the proportion of males over the total number of sexed nestlings in each particular nucleus and year. 95% confidence intervals and sample sizes are shown in brackets. In bold, values of observed sex ratios that fall outside the 95% confidence interval obtained in the simulations, and underlined, values that lie on the limit of the 95% confidence interval.

|  | **high-altitude area** | | | | **low-altitude area** | | |
| --- | --- | --- | --- | --- | --- | --- | --- |
|  | Madrid nucleus | Segovia nucleus  A | Segovia nucleus  B | Segovia nucleus  C | Ávila nucleus  A  (eastern Gredos) | Ávila nucleus  B | Ávila nucleus  C |
| 2004 | 0.61  (0.33-0.67; n=36) |  |  |  |  |  |  |
| 2005 |  | 0.50  (0.00-1.00; n=2) | 0.40  (0.00-0.80; n=5) | 0.67  (0.17-0.83; n=6) |  |  |  |
| 2007 |  |  | 0.67  (0.00-1.00; n=3) | 0.17  (0.17-0.83; n=6) | 0.33  (0.00-1.00; n=3) | 0.50  (0.00-1.00; n=4) |  |
| 2010 | 0.59  (0.29-0.76; n=17) |  |  | 0.67  (0.00-1.00; n=3) | 0.50  (0.00-1.00; n=2) |  |  |
| 2013 |  |  |  |  | 0.40  (0.00-0.80; n=5) |  |  |
| 2014 |  |  |  | 1.00  (0.00-1.00; n=2) | 0.50  (0.00-1.00; n=2) |  |  |
| 2015 |  |  |  | 0.50  (0.00-1.00; n=4) | 0.50  (0.00-1.00; n=6) |  |  |
| 2016 |  | 0.50  (0.00-1.00; n=2) |  | 1.00  (0.00-1.00; n=2) | 0.70  (0.20-0.80; n=10) |  |  |
| 2017 |  | 0.00  (0.00-1.00; n=2) |  | 0.50  (0.00-1.00; n=2) | 0.25  (0.25-0.75; n=12) |  |  |
| 2018 |  |  |  |  | 0.33  (0.17-0.83; n=6) |  |  |
| 2019 |  |  |  |  | 0.67  (0.00-1.00; n=3) |  |  |
| 2020 |  |  |  |  | **1.00**  **(0.20-0.80; n=10)** |  |  |
| 2021 |  |  |  |  | 0.38  (0.13-0.88; n=8) |  |  |
| 2022 |  |  |  |  | 0.50  (0.00-1.00; n=4) |  |  |
| 2023 |  |  |  |  | 0.60  (0.20-0.80; n=10) |  | 0.67  (0.22-0.78; n=9) |

**Table S2**. Models assessing the effects of food availability (estimated based on the restrictive/post-restrictive periods of the mad-cow crisis; mad-cows) and hatching date on the probability of a nestling cinereous vulture *Aegypius monachus* being a male in the low- and high-altitude areas separately. Low-altitude area: models including (A.1) all sexed nestlings (n = 94) and (A.2) only sexed nestlings of known hatching date (n = 93). High-altitude area: models including (B.1) all sexed nestlings (n = 92) and (B.2) only sexed nestlings of known hatching date (n = 74). Estimates, standard errors (SE), and 95% confidence intervals (CI) are shown for alternative models only (ΔAICc < 2). Year was included as a random term in all models. The null model was included in all sets of models. In bold, significant effects (i.e., the 95% CI of the estimate does not overlap zero). df: degrees of freedom; AICc: Akaike information criterion corrected for small sample sizes; ΔAICc: difference between the AICc of model *i* and that of the best model (i.e. the model with the lowest AICc); w: Akaike weight.

| **Model selection** | |  |  | |  |  |
| --- | --- | --- | --- | --- | --- | --- |
| **Model** | | **df** | **AICc** | | **ΔAICc** | **w** |
| (A.1) low-altitude area | |  |  | |  |  |
| null | | 2 | 131.54 | | 0.00 | 0.72 |
| mad-cows | | 3 | 133.46 | | 1.92 | 0.28 |
| (A.2) low-altitude area | |  |  | |  |  |
| null | | 2 | 130.48 | | 0.00 | 0.73 |
| hatching date | | 3 | 132.45 | | 1.97 | 0.27 |
| (B.1) high-altitude area | |  |  | |  |  |
| null | | 2 | 130.10 | | 0.00 | 0.74 |
| mad-cows | | 3 | 132.24 | | 2.14 | 0.26 |
| (B.2) high-altitude area | |  |  | |  |  |
| hatching date | | 3 | 103.74 | | 0.00 | 0.75 |
| null | | 2 | 105.88 | | 2.15 | 0.25 |
| **Variable** | **Estimate** | | | **SE** | **2.5% CI** | **97.5% CI** |
| (A.1) |  | | |  |  |  |
| mad-cows (post) | 0.41 | | | 0.89 | -1.32 | 2.15 |
| (A.2) |  | | |  |  |  |
| hatching date | -0.10 | | | 0.24 | -0.58 | 0.38 |
| (B.2) |  | | |  |  |  |
| **hatching date** | **0.48** | | | **0.24** | **0.00** | **0.96** |

**Table S3**. Models assessing the effects of temperature (mean minimum temperature: min.mean; mean maximum temperature: max.mean; and mean average temperature: avg.mean) over different periods before the fertilization date on the probability of a nestling cinereous vulture *Aegypius monachus* being a male in the low- and high-altitude areas separately (see Materials and methods for details). (A) Models including nestlings with temperature data available in the low- (n = 86) and (B) high-altitude areas (n = 70). Estimates, standard errors (SE), and 95% confidence intervals (CI) are shown for alternative models only (ΔAICc < 2). Year was included as a random term in all models. The null model was included in both sets of models. In bold, significant effects (i.e., the 95% CI of the estimate does not overlap zero). df: degrees of freedom; AICc: Akaike information criterion corrected for small sample sizes; ΔAICc: difference between the AICc of model *i* and that of the best model (i.e. the model with the lowest AICc); w: Akaike weight.

| **Model selection** |  |  |  |  |
| --- | --- | --- | --- | --- |
| **Model** | **df** | **AICc** | **ΔAICc** | **w** |
| (A) low-altitude area |  |  |  |  |
| max.mean (30-90) | 3 | 119.64 | 0.00 | 0.07 |
| avg.mean (60-90) | 3 | 119.86 | 0.22 | 0.07 |
| avg.mean (30-90) | 3 | 120.10 | 0.46 | 0.06 |
| null | 2 | 120.22 | 0.57 | 0.06 |
| max.mean (0-90) | 3 | 120.25 | 0.61 | 0.05 |
| max.mean (60-90) | 3 | 120.39 | 0.75 | 0.05 |
| min.mean (60-90) | 3 | 120.42 | 0.78 | 0.05 |
| min.mean (30-90) | 3 | 121.09 | 1.44 | 0.04 |
| max.mean (30-60) | 3 | 121.24 | 1.60 | 0.03 |
| max.mean (0-60) | 3 | 121.28 | 1.64 | 0.03 |
| avg.mean (0-90) | 3 | 121.30 | 1.66 | 0.03 |
| max.mean (0-120) | 3 | 121.39 | 1.74 | 0.03 |
| min.mean (60-120) | 3 | 121.66 | 2.02 | 0.03 |
| avg.mean (30-120) | 3 | 121.70 | 2.06 | 0.03 |
| max.mean (30-120) | 3 | 121.71 | 2.07 | 0.03 |
| min.mean (30-120) | 3 | 121.79 | 2.15 | 0.03 |
| avg.mean (0-120) | 3 | 121.80 | 2.16 | 0.03 |
| avg.mean (30-60) | 3 | 121.81 | 2.17 | 0.03 |
| avg.mean (60-120) | 3 | 121.90 | 2.26 | 0.02 |
| max.mean (60-120) | 3 | 122.13 | 2.49 | 0.02 |
| avg.mean (0-60) | 3 | 122.15 | 2.50 | 0.02 |
| min.mean (0-90) | 3 | 122.16 | 2.52 | 0.02 |
| min.mean (0-120) | 3 | 122.16 | 2.52 | 0.02 |
| min.mean (0-30) | 3 | 122.25 | 2.61 | 0.02 |
| min.mean (30-60) | 3 | 122.25 | 2.61 | 0.02 |
| max.mean (0-30) | 3 | 122.25 | 2.61 | 0.02 |
| min.mean (90-120) | 3 | 122.26 | 2.62 | 0.02 |
| avg.mean (90-120) | 3 | 122.35 | 2.71 | 0.02 |
| max.mean (90-120) | 3 | 122.35 | 2.71 | 0.02 |
| min.mean (0-60) | 3 | 122.36 | 2.72 | 0.02 |
| avg.mean (0-30) | 3 | 122.36 | 2.72 | 0.02 |
| (B) high-altitude area |  |  |  |  |
| min.mean (60-120) | 3 | 95.70 | 0.00 | 0.34 |
| min.mean (90-120) | 3 | 96.97 | 1.27 | 0.18 |
| avg.mean (90-120) | 3 | 99.90 | 4.20 | 0.04 |
| null | 2 | 100.30 | 4.60 | 0.03 |
| min.mean (60-90) | 3 | 100.41 | 4.70 | 0.03 |
| min.mean (30-120) | 3 | 100.58 | 4.87 | 0.03 |
| min.mean (0-120) | 3 | 100.67 | 4.97 | 0.03 |
| min.mean (30-60) | 3 | 101.02 | 5.31 | 0.02 |
| avg.mean (60-120) | 3 | 101.32 | 5.62 | 0.02 |
| max.mean (90-120) | 3 | 101.78 | 6.08 | 0.02 |
| avg.mean (30-60) | 3 | 101.79 | 6.09 | 0.02 |
| min.mean (0-30) | 3 | 101.90 | 6.20 | 0.02 |
| max.mean (60-90) | 3 | 102.04 | 6.34 | 0.01 |
| max.mean (30-90) | 3 | 102.10 | 6.40 | 0.01 |
| max.mean (0-90) | 3 | 102.20 | 6.49 | 0.01 |
| max.mean (30-60) | 3 | 102.23 | 6.53 | 0.01 |
| max.mean (0-60) | 3 | 102.27 | 6.56 | 0.01 |
| avg.mean (30-90) | 3 | 102.27 | 6.57 | 0.01 |
| max.mean (0-30) | 3 | 102.33 | 6.63 | 0.01 |
| avg.mean (0-60) | 3 | 102.34 | 6.64 | 0.01 |
| avg.mean (30-120) | 3 | 102.34 | 6.64 | 0.01 |
| min.mean (0-90) | 3 | 102.35 | 6.65 | 0.01 |
| avg.mean (0-90) | 3 | 102.41 | 6.71 | 0.01 |
| avg.mean (0-120) | 3 | 102.42 | 6.72 | 0.01 |
| max.mean (0-120) | 3 | 102.44 | 6.74 | 0.01 |
| min.mean (0-60) | 3 | 102.46 | 6.76 | 0.01 |
| max.mean (60-120) | 3 | 102.47 | 6.76 | 0.01 |
| max.mean (30-120) | 3 | 102.48 | 6.78 | 0.01 |
| min.mean (30-90) | 3 | 102.48 | 6.78 | 0.01 |
| avg.mean (60-90) | 3 | 102.49 | 6.78 | 0.01 |
| avg.mean (0-30) | 3 | 102.49 | 6.79 | 0.01 |
| **Variable** | **Estimate** | **SE** | **2.5% CI** | **97.5% CI** |
| (A) |  |  |  |  |
| max.mean (30-90) | 0.42 | 0.25 | -0.07 | 0.91 |
| avg.mean (60-90) | 0.40 | 0.25 | -0.09 | 0.90 |
| avg.mean (30-90) | 0.43 | 0.28 | -0.12 | 0.99 |
| max.mean (0-90) | 0.39 | 0.25 | -0.10 | 0.88 |
| max.mean (60-90) | 0.31 | 0.21 | -0.11 | 0.72 |
| min.mean (60-90) | 0.33 | 0.24 | -0.14 | 0.80 |
| min.mean (30-90) | 0.32 | 0.28 | -0.23 | 0.87 |
| max.mean (30-60) | 0.19 | 0.18 | -0.16 | 0.53 |
| max.mean (0-60) | 0.24 | 0.22 | -0.19 | 0.68 |
| avg.mean (0-90) | 0.30 | 0.28 | -0.25 | 0.85 |
| max.mean (0-120) | 0.28 | 0.28 | -0.26 | 0.82 |
| (B) |  |  |  |  |
| **min.mean (60-120)** | **-0.61** | **0.26** | **-1.11** | **-0.11** |
| **min.mean (90-120)** | **-0.33** | **0.15** | **-0.62** | **-0.04** |

**Table S4**. Models assessing the effects of climatic variables (mean minimum temperature: min.mean; standard deviation of the mean minimum temperature: min.sd; range of the mean minimum temperature: min.range; mean maximum temperature: max.mean; standard deviation of the mean maximum temperature: max.sd; range of the mean maximum temperature: max.range; mean average temperature: avg.mean; and total rainfall: rainfall) over different periods before the fertilization date on the probability of a nestling cinereous vulture *Aegypius monachus* being a male (see Materials and methods for details). (A) Models including nestlings with temperature data available (n = 156); (B) models including nestlings with rainfall data available (n = 139). Estimates, standard errors (SE), and 95% confidence intervals (CI) are shown for alternative models only (ΔAICc < 2). Year was included as a random term in all models. The null model was included in both sets of models. In bold, significant effects (i.e., the 95% CI of the estimate does not overlap zero). df: degrees of freedom; AICc: Akaike information criterion corrected for small sample sizes; ΔAICc: difference between the AICc of model *i* and that of the best model (i.e. the model with the lowest AICc); w: Akaike weight.

| **Model selection** |  |  |  |  |
| --- | --- | --- | --- | --- |
| **Model** | **df** | **AICc** | **ΔAICc** | **w** |
| (A) temperature |  |  |  |  |
| min.sd (90-120) | 3 | 215.76 | 0.00 | 0.06 |
| min.mean (60-120)*area | 5 | 215.94 | 0.18 | 0.05 |
| null | 2 | 216.67 | 0.91 | 0.04 |
| max.sd (60-90) | 3 | 217.30 | 1.54 | 0.03 |
| min.mean (90-120) | 3 | 217.66 | 1.90 | 0.02 |
| min.range (0-30) | 3 | 217.80 | 2.04 | 0.02 |
| avg.mean (90-120) | 3 | 217.89 | 2.13 | 0.02 |
| min.mean (90-120)*area | 5 | 217.90 | 2.14 | 0.02 |
| min.range (90-120) | 3 | 218.11 | 2.35 | 0.02 |
| max.mean (90-120) | 3 | 218.13 | 2.37 | 0.02 |
| min.sd (0-30) | 3 | 218.18 | 2.42 | 0.02 |
| max.mean (30-90) | 3 | 218.28 | 2.52 | 0.02 |
| min.mean (60-120) | 3 | 218.29 | 2.53 | 0.02 |
| max.mean (60-90) | 3 | 218.29 | 2.53 | 0.02 |
| avg.mean (30-60) | 3 | 218.38 | 2.62 | 0.01 |
| min.mean (0-30) | 3 | 218.39 | 2.63 | 0.01 |
| min.mean (60-90)*area | 5 | 218.40 | 2.64 | 0.01 |
| max.mean (30-60) | 3 | 218.40 | 2.64 | 0.01 |
| max.sd (30-60) | 3 | 218.44 | 2.68 | 0.01 |
| min.mean (30-60) | 3 | 218.44 | 2.68 | 0.01 |
| max.mean (0-90) | 3 | 218.46 | 2.71 | 0.01 |
| min.sd (30-60) | 3 | 218.48 | 2.72 | 0.01 |
| avg.mean (30-90) | 3 | 218.50 | 2.74 | 0.01 |
| max.range (60-90) | 3 | 218.50 | 2.74 | 0.01 |
| max.range (0-30) | 3 | 218.55 | 2.79 | 0.01 |
| min.mean (0-120) | 3 | 218.55 | 2.80 | 0.01 |
| max.mean (0-60) | 3 | 218.56 | 2.80 | 0.01 |
| avg.mean (60-120) | 3 | 218.56 | 2.80 | 0.01 |
| max.sd (90-120) | 3 | 218.57 | 2.81 | 0.01 |
| min.range (30-60) | 3 | 218.58 | 2.82 | 0.01 |
| min.range (60-90) | 3 | 218.59 | 2.83 | 0.01 |
| min.mean (30-120) | 3 | 218.64 | 2.88 | 0.01 |
| avg.mean (60-90) | 3 | 218.65 | 2.89 | 0.01 |
| max.range (30-60) | 3 | 218.65 | 2.89 | 0.01 |
| max.sd (0-30) | 3 | 218.66 | 2.90 | 0.01 |
| avg.mean (0-90) | 3 | 218.69 | 2.93 | 0.01 |
| min.mean (30-90) | 3 | 218.69 | 2.93 | 0.01 |
| max.mean (0-30) | 3 | 218.69 | 2.93 | 0.01 |
| max.mean (60-120) | 3 | 218.70 | 2.95 | 0.01 |
| avg.mean (0-60) | 3 | 218.71 | 2.95 | 0.01 |
| avg.mean (0-30) | 3 | 218.72 | 2.96 | 0.01 |
| avg.mean (0-120) | 3 | 218.73 | 2.97 | 0.01 |
| min.mean (0-60) | 3 | 218.73 | 2.97 | 0.01 |
| min.mean (0-90) | 3 | 218.73 | 2.97 | 0.01 |
| avg.mean (30-120) | 3 | 218.73 | 2.97 | 0.01 |
| max.mean (0-120) | 3 | 218.73 | 2.97 | 0.01 |
| min.sd (60-90) | 3 | 218.73 | 2.97 | 0.01 |
| area | 3 | 218.74 | 2.98 | 0.01 |
| min.mean (60-90) | 3 | 218.74 | 2.98 | 0.01 |
| max.mean (30-120) | 3 | 218.74 | 2.98 | 0.01 |
| max.range (90-120) | 3 | 218.75 | 2.99 | 0.01 |
| min.range (0-30)*area | 5 | 219.11 | 3.35 | 0.01 |
| min.mean(30-120)*area | 5 | 219.73 | 3.97 | 0.01 |
| min.sd (90-120)*area | 5 | 219.74 | 3.98 | 0.01 |
| min.sd (0-30)*area | 5 | 219.74 | 3.98 | 0.01 |
| max.mean (30-90)*area | 5 | 219.81 | 4.05 | 0.01 |
| min.range (30-60)*area | 5 | 220.01 | 4.25 | 0.01 |
| avg.mean (60-90)*area | 5 | 220.07 | 4.31 | 0.01 |
| avg.mean (90-120)*area | 5 | 220.09 | 4.33 | 0.01 |
| min.mean (0-120)*area | 5 | 220.25 | 4.49 | 0.01 |
| max.mean (0-90)*area | 5 | 220.36 | 4.60 | 0.01 |
| avg.mean (30-90)*area | 5 | 220.45 | 4.69 | 0.01 |
| max.mean (60-90)*area | 5 | 220.56 | 4.80 | 0.01 |
| avg.mean (60-120)*area | 5 | 220.59 | 4.83 | 0.00 |
| min.sd (30-60)*area | 5 | 220.84 | 5.08 | 0.00 |
| max.sd (0-30)*area | 5 | 221.07 | 5.31 | 0.00 |
| max.range (0-30)*area | 5 | 221.10 | 5.34 | 0.00 |
| max.sd (60-90)*area | 5 | 221.14 | 5.38 | 0.00 |
| min.mean (30-90)*area | 5 | 221.49 | 5.73 | 0.00 |
| max.mean (0-60)*area | 5 | 221.50 | 5.74 | 0.00 |
| max.mean (30-60)*area | 5 | 221.60 | 5.84 | 0.00 |
| avg.mean (30-120)*area | 5 | 221.60 | 5.84 | 0.00 |
| max.mean (90-120)*area | 5 | 221.65 | 5.89 | 0.00 |
| avg.mean (0-90)*area | 5 | 221.71 | 5.95 | 0.00 |
| max.mean (0-120)*area | 5 | 221.84 | 6.08 | 0.00 |
| max.sd (30-60)*area | 5 | 221.88 | 6.13 | 0.00 |
| avg.mean (0-120)*area | 5 | 221.91 | 6.15 | 0.00 |
| avg.mean (30-60)*area | 5 | 222.04 | 6.28 | 0.00 |
| min.mean (30-60)*area | 5 | 222.05 | 6.29 | 0.00 |
| max.mean (30-120)*area | 5 | 222.18 | 6.43 | 0.00 |
| min.mean (0-30)*area | 5 | 222.23 | 6.47 | 0.00 |
| min.mean (0-90)*area | 5 | 222.26 | 6.50 | 0.00 |
| min.range (90-120)*area | 5 | 222.33 | 6.57 | 0.00 |
| max.mean (60-120)*area | 5 | 222.42 | 6.66 | 0.00 |
| max.range (60-90)*area | 5 | 222.44 | 6.68 | 0.00 |
| max.range (30-60)*area | 5 | 222.59 | 6.83 | 0.00 |
| avg.mean (0-60)*area | 5 | 222.65 | 6.89 | 0.00 |
| min.range (60-90)*area | 5 | 222.66 | 6.90 | 0.00 |
| max.mean (0-30)*area | 5 | 222.74 | 6.98 | 0.00 |
| max.sd (90-120)*area | 5 | 222.76 | 7.00 | 0.00 |
| min.sd (60-90)*area | 5 | 222.89 | 7.13 | 0.00 |
| avg.mean (0-30)*area | 5 | 222.92 | 7.16 | 0.00 |
| max.range (90-120)*area | 5 | 222.96 | 7.21 | 0.00 |
| min.mean (0-60)*area | 5 | 222.97 | 7.21 | 0.00 |
| (B) rainfall |  |  |  |  |
| null | 2 | 196.52 | 0.00 | 0.13 |
| rainfall (30-60) | 3 | 196.53 | 0.01 | 0.13 |
| rainfall (30-90) | 3 | 196.75 | 0.23 | 0.11 |
| rainfall (90-120) | 3 | 197.93 | 1.41 | 0.06 |
| rainfall (30-60)*area | 5 | 197.94 | 1.41 | 0.06 |
| rainfall (0-90) | 3 | 198.09 | 1.57 | 0.06 |
| rainfall (60-90) | 3 | 198.22 | 1.69 | 0.05 |
| rainfall (0-60) | 3 | 198.32 | 1.79 | 0.05 |
| area | 3 | 198.44 | 1.92 | 0.05 |
| rainfall (60-120) | 3 | 198.50 | 1.97 | 0.05 |
| rainfall (0-30) | 3 | 198.53 | 2.01 | 0.05 |
| rainfall (30-120) | 3 | 198.56 | 2.03 | 0.05 |
| rainfall (0-120) | 3 | 198.61 | 2.08 | 0.04 |
| rainfall (0-60)*area | 5 | 199.30 | 2.78 | 0.03 |
| rainfall (30-90)*area | 5 | 200.36 | 3.84 | 0.02 |
| rainfall (90-120)*area | 5 | 200.82 | 4.30 | 0.01 |
| rainfall (60-120)*area | 5 | 201.16 | 4.64 | 0.01 |
| rainfall (0-90)*area | 5 | 201.26 | 4.74 | 0.01 |
| rainfall (0-30)*area | 5 | 201.64 | 5.12 | 0.01 |
| rainfall (60-90)*area | 5 | 202.35 | 5.83 | 0.01 |
| rainfall (0-120)*area | 5 | 202.48 | 5.95 | 0.01 |
| rainfall (30-120)*area | 5 | 202.70 | 6.18 | 0.01 |
| **Variable** | **Estimate** | **SE** | **2.5% CI** | **97.5% CI** |
| (A) |  |  |  |  |
| min.sd (90-120) | 0.53 | 0.31 | -0.08 | 1.15 |
| min.mean (60-120) | 0.16 | 0.20 | -0.23 | 0.55 |
| area (high-altitude) | 1.53 | 1.11 | -0.64 | 3.70 |
| **min.mean (60-120)*area (high-altitude)** | **-0.77** | **0.33** | **-1.41** | **-0.13** |
| max.sd (60-90) | -0.35 | 0.29 | -0.93 | 0.23 |
| min.mean (90-120) | -0.08 | 0.08 | -0.23 | 0.07 |
| (B) |  |  |  |  |
| rainfall (30-60) | 0.25 | 0.17 | -0.09 | 0.59 |
| rainfall (30-90) | 0.24 | 0.17 | -0.10 | 0.58 |
| rainfall (90-120) | -0.15 | 0.18 | -0.49 | 0.20 |
| rainfall (30-60) | -0.17 | 0.39 | -0.94 | 0.61 |
| area (high-altitude) | -0.16 | 0.47 | -1.07 | 0.75 |
| rainfall (30-60)*area (high-altitude) | 0.76 | 0.49 | -0.19 | 1.72 |
| rainfall (0-90) | 0.13 | 0.18 | -0.22 | 0.49 |
| rainfall (60-90) | 0.11 | 0.18 | -0.23 | 0.46 |
| rainfall (0-60) | 0.10 | 0.19 | -0.26 | 0.46 |
| area (high-altitude) | 0.15 | 0.36 | -0.55 | 0.86 |
| rainfall (60-120) | -0.06 | 0.18 | -0.42 | 0.30 |

**Table S5**. Models assessing the effects of climatic variables (mean minimum temperature: min.mean; mean maximum temperature: max.mean; mean average temperature: avg.mean; and total rainfall: rainfall) over each winter before fertilization on the annual offspring sex ratio of cinereous vultures *Aegypius monachus* in eastern Gredos (n = 67 nestlings) (see Materials and methods for details). Estimates, standard errors (SE), and 95% confidence intervals (CI) are shown for alternative models only (ΔAICc<2). The null model was included in our set of models. In bold, significant effects (i.e., the 95% CI of the estimate does not overlap zero). df: degrees of freedom; AICc: Akaike information criterion corrected for small sample sizes; ΔAICc: difference between the AICc of model *i* and that of the best model (i.e. the model with the lowest AICc); w: Akaike weight.

| **Model selection** |  |  |  |  |
| --- | --- | --- | --- | --- |
| **Model** | **df** | **AICc** | **ΔAICc** | **w** |
| avg.mean | 2 | 29.64 | 0.00 | 0.24 |
| min.mean | 2 | 29.76 | 0.11 | 0.23 |
| max.mean | 2 | 30.55 | 0.91 | 0.15 |
| null | 1 | 30.88 | 1.24 | 0.13 |
| min.range | 2 | 32.21 | 2.56 | 0.07 |
| rainfall | 2 | 33.46 | 3.81 | 0.04 |
| max.sd | 2 | 33.56 | 3.92 | 0.03 |
| max.range | 2 | 34.07 | 4.42 | 0.03 |
| min.sd | 2 | 34.22 | 4.57 | 0.02 |
| min.mean + rainfall | 3 | 34.41 | 4.76 | 0.02 |
| avg.mean + rainfall | 3 | 34.81 | 5.16 | 0.02 |
| max.mean + min.mean | 3 | 35.08 | 5.43 | 0.02 |
| max.mean + rainfall | 3 | 35.87 | 6.23 | 0.01 |
| min.mean*rainfall | 4 | 43.34 | 13.69 | 0.00 |
| max.mean + min.mean + rainfall | 4 | 43.70 | 14.06 | 0.00 |
| max.mean*min.mean | 4 | 43.95 | 14.30 | 0.00 |
| avg.mean*rainfall | 4 | 44.13 | 14.48 | 0.00 |
| max.mean*rainfall | 4 | 45.20 | 15.56 | 0.00 |
| **Variable** | **Estimate** | **SE** | **2.5% CI** | **97.5% CI** |
| **avg.mean** | **0.67** | **0.31** | **0.05** | **1.28** |
| **min.mean** | **0.66** | **0.31** | **0.04** | **1.28** |
| max.mean | 0.61 | 0.32 | -0.01 | 1.23 |

**Table S6**. Models assessing the effects of temperature (mean minimum temperature: min.mean; mean maximum temperature: max.mean; and mean average temperature: avg.mean) over different periods before the fertilization date on the probability of a nestling cinereous vulture *Aegypius monachus* being a male in eastern Gredos (n = 77 nestlings) (see Materials and methods for details). Estimates, standard errors (SE), and 95% confidence intervals (CI) are shown for alternative models only (ΔAICc<2). Year was included as a random term in all models. The null model was included in our set of models. In bold, significant effects (i.e., the 95% of the estimate CI does not overlap zero). df: degrees of freedom; AICc: Akaike information criterion corrected for small sample sizes; ΔAICc: difference between the AICc of model *i* and that of the best model (i.e. the model with the lowest AICc); w: Akaike weight.

| **Model selection** | |  |  |  |  |
| --- | --- | --- | --- | --- | --- |
| **Model** | | **df** | **AICc** | **ΔAICc** | **w** |
| min.mean (0-90) | | 3 | 106.23 | 0.00 | 0.09 |
| avg.mean (0-90) | | 3 | 106.24 | 0.01 | 0.09 |
| avg.mean (30-90) | | 3 | 106.82 | 0.60 | 0.07 |
| min.mean (30-90) | | 3 | 107.18 | 0.95 | 0.06 |
| max.mean (0-90) | | 3 | 107.31 | 1.08 | 0.05 |
| max.mean (30-90) | | 3 | 107.33 | 1.10 | 0.05 |
| avg.mean (60-90) | | 3 | 107.40 | 1.17 | 0.05 |
| min.mean (60-90) | | 3 | 107.80 | 1.57 | 0.04 |
| null | | 2 | 108.19 | 1.96 | 0.04 |
| max.mean (60-90) | | 3 | 108.23 | 2.00 | 0.03 |
| min.mean (0-120) | | 3 | 108.45 | 2.22 | 0.03 |
| avg.mean (0-60) | | 3 | 108.48 | 2.25 | 0.03 |
| avg.mean (0-120) | | 3 | 108.49 | 2.27 | 0.03 |
| max.mean (0-60) | | 3 | 108.60 | 2.37 | 0.03 |
| max.mean (0-120) | | 3 | 108.93 | 2.70 | 0.02 |
| min.mean (30-120) | | 3 | 109.03 | 2.80 | 0.02 |
| max.mean (30-60) | | 3 | 109.11 | 2.88 | 0.02 |
| avg.mean (30-60) | | 3 | 109.18 | 2.95 | 0.02 |
| min.mean (0-60) | | 3 | 109.25 | 3.02 | 0.02 |
| avg.mean (30-120) | | 3 | 109.34 | 3.11 | 0.02 |
| min.mean (60-120) | | 3 | 109.35 | 3.12 | 0.02 |
| max.mean (30-120) | | 3 | 109.65 | 3.42 | 0.02 |
| min.mean (30-60) | | 3 | 109.70 | 3.47 | 0.02 |
| avg.mean (60-120) | | 3 | 109.78 | 3.55 | 0.02 |
| max.mean (0-30) | | 3 | 110.05 | 3.82 | 0.01 |
| max.mean (60-120) | | 3 | 110.11 | 3.88 | 0.01 |
| avg.mean (0-30) | | 3 | 110.14 | 3.91 | 0.01 |
| min.mean (90-120) | | 3 | 110.16 | 3.94 | 0.01 |
| min.mean (0-30) | | 3 | 110.31 | 4.08 | 0.01 |
| avg.mean (90-120) | | 3 | 110.34 | 4.11 | 0.01 |
| max.mean (90-120) | | 3 | 110.35 | 4.12 | 0.01 |
| **Variable** | **Estimate** | | **SE** | **2.5% CI** | **97.5% CI** |
| **min.mean (0-90)** | **1.06** | | **0.48** | **0.12** | **2.01** |
| **avg.mean (0-90)** | **0.79** | | **0.34** | **0.12** | **1.46** |
| avg.mean (30-90) | 0.62 | | 0.32 | 0.00 | 1.25 |
| min.mean (30-90) | 0.66 | | 0.36 | -0.05 | 1.38 |
| max.mean (0-90) | 0.50 | | 0.26 | 0.00 | 1.01 |
| max.mean (30-90) | 0.47 | | 0.26 | -0.03 | 0.98 |
| avg.mean (60-90) | 0.46 | | 0.27 | -0.06 | 0.99 |
| min.mean (60-90) | 0.42 | | 0.27 | -0.11 | 0.95 |

**Table S7**. Models assessing the effects of total rainfall over two periods after the fertilization date on the probability of a nestling cinereous vulture *Aegypius monachus* being a male in the low- and high-altitude areas separately (see Materials and methods for details). (A) Models including nestlings with rainfall data available in the low- (n = 86) and (B) high-altitude areas (n = 70). Estimates, standard errors (SE), and 95% confidence intervals (CI) are shown for alternative models only (ΔAICc < 2). Year was included as a random term in all models. The null model was included in both sets of models. df: degrees of freedom; AICc: Akaike information criterion corrected for small sample sizes; ΔAICc: difference between the AICc of model *i* and that of the best model (i.e. the model with the lowest AICc); w: Akaike weight.

| **Model selection** |  |  |  |  |
| --- | --- | --- | --- | --- |
| **Model** | **df** | **AICc** | **ΔAICc** | **w** |
| (A) low-altitude area |  |  |  |  |
| rainfall (hatch-band) | 3 | 119.29 | 0.00 | 0.54 |
| null | 2 | 120.22 | 0.93 | 0.34 |
| rainfall (fert-hatch) | 3 | 122.19 | 2.90 | 0.13 |
| (B) high-altitude area |  |  |  |  |
| rainfall (hatch-band) | 3 | 98.79 | 0.00 | 0.60 |
| null | 2 | 100.30 | 1.52 | 0.28 |
| rainfall (fert-hatch) | 3 | 102.12 | 3.33 | 0.11 |
| **Variable** | **Estimate** | **SE** | **2.5% CI** | **97.5% CI** |
| (A) |  |  |  |  |
| rainfall (hatch-band) | 0.65 | 0.41 | -0.14 | 1.45 |
| (B) |  |  |  |  |
| rainfall (hatch-band) | -0.43 | 0.23 | -0.88 | 0.02 |

**Table S8**. Models assessing the effects of the breeding area (area) and climatic variables (mean minimum temperature: min.mean; standard deviation of the mean minimum temperature: min.sd; range of the mean minimum temperature: min.range; mean maximum temperature: max.mean; standard deviation of the mean maximum temperature: max.sd; range of the mean maximum temperature: max.range; mean average temperature: avg.mean; and total rainfall: rainfall) over two periods after the fertilization date on the probability of a nestling cinereous vulture *Aegypius monachus* being a male (n = 156 nestlings) (see Materials and methods for details). Estimates, standard errors (SE), and 95% confidence intervals (CI) are shown. Year was included as a random term in all models. The null model was included in both sets of models. In bold, significant effects (i.e., the 95% CI of the estimate does not overlap zero). df: degrees of freedom; AICc: Akaike information criterion corrected for small sample sizes; ΔAICc: difference between the AICc of model *i* and that of the best model (i.e. the model with the lowest AICc); w: Akaike weight.

| **Model selection** | |  |  |  |  |
| --- | --- | --- | --- | --- | --- |
| **Model** | | **df** | **AICc** | **ΔAICc** | **w** |
| rainfall (hatch-band)*area | | 5 | 216.21 | 0.00 | 0.12 |
| null | | 2 | 216.67 | 0.46 | 0.10 |
| max.range (hatch-band) | | 3 | 217.92 | 1.71 | 0.05 |
| max.range (fert-hatch) | | 3 | 218.05 | 1.84 | 0.05 |
| min.range (hatch-band) | | 3 | 218.20 | 1.99 | 0.04 |
| min.sd (hatch-band) | | 3 | 218.24 | 2.03 | 0.04 |
| rainfall (hatch-band) | | 3 | 218.41 | 2.20 | 0.04 |
| max.sd (hatch-band) | | 3 | 218.44 | 2.22 | 0.04 |
| rainfall (fert-hatch) | | 3 | 218.51 | 2.30 | 0.04 |
| min.sd (fert-hatch) | | 3 | 218.58 | 2.37 | 0.04 |
| max.sd (fert-hatch) | | 3 | 218.61 | 2.40 | 0.04 |
| avg.mean (fert-hatch) | | 3 | 218.66 | 2.45 | 0.04 |
| min.mean (fert-hatch) | | 3 | 218.66 | 2.45 | 0.04 |
| min.range (fert-hatch) | | 3 | 218.66 | 2.45 | 0.04 |
| max.mean (fert-hatch) | | 3 | 218.67 | 2.46 | 0.04 |
| max.mean (hatch-band) | | 3 | 218.72 | 2.51 | 0.03 |
| min.mean (hatch-band) | | 3 | 218.72 | 2.51 | 0.03 |
| area | | 3 | 218.74 | 2.52 | 0.03 |
| avg.mean (hatch-band) | | 3 | 218.75 | 2.53 | 0.03 |
| max.range (fert-hatch)*area | | 5 | 219.63 | 3.42 | 0.02 |
| max.sd (fert-hatch)*area | | 5 | 219.82 | 3.61 | 0.02 |
| min.sd (fert-hatch)*area | | 5 | 221.08 | 4.87 | 0.01 |
| avg.mean (fert-hatch)*area | | 5 | 221.33 | 5.12 | 0.01 |
| min.mean (fert-hatch)*area | | 5 | 221.36 | 5.15 | 0.01 |
| max.mean (fert-hatch)*area | | 5 | 221.44 | 5.23 | 0.01 |
| max.mean (hatch-band)*area | | 5 | 221.62 | 5.41 | 0.01 |
| avg.mean (hatch-band)*area | | 5 | 221.96 | 5.75 | 0.01 |
| max.range (hatch-band)*area | | 5 | 222.08 | 5.87 | 0.01 |
| min.range (fert-hatch)*area | | 5 | 222.18 | 5.97 | 0.01 |
| min.range (hatch-band)*area | | 5 | 222.21 | 6.00 | 0.01 |
| min.sd (hatch-band)*area | | 5 | 222.48 | 6.26 | 0.01 |
| min.mean (hatch-band)*area | | 5 | 222.50 | 6.29 | 0.01 |
| max.sd (hatch-band)*area | | 5 | 222.51 | 6.30 | 0.01 |
| rainfall (fert-hatch)*area | | 5 | 222.70 | 6.49 | 0.00 |
| **Variable** | **Estimate** | | **SE** | **2.5% CI** | **97.5% CI** |
| rainfall (hatch-band) | 0.59 | | 0.37 | -0.12 | 1.31 |
| area (high altitude) | -0.08 | | 0.47 | -0.99 | 0.84 |
| **rainfall (hatch-band)*area (high altitude)** | **-1.06** | | **0.44** | **-1.92** | **-0.19** |
| max.range (hatch-band) | -0.05 | | 0.06 | -0.17 | 0.06 |
| max.range (fert-hatch) | 0.05 | | 0.06 | -0.07 | 0.17 |
| min.range (hatch-band) | -0.05 | | 0.07 | -0.19 | 0.08 |

**Table S9**. Models assessing the effects of climatic variables (total rainfall: rainfall; mean minimum temperature: min.mean; mean maximum temperature: max.mean; and mean average temperature: avg.mean) over two periods after the fertilization date on the probability of a nestling cinereous vulture *Aegypius monachus* being a male in eastern Gredos (n = 77 nestlings) (see Materials and methods for details). Estimates, standard errors (SE), and 95% confidence intervals (CI) are shown for alternative models only (ΔAICc<2). Year was included as a random term in all models. The null model was included in our set of models. df: degrees of freedom; AICc: Akaike information criterion corrected for small sample sizes; ΔAICc: difference between the AICc of model *i* and that of the best model (i.e. the model with the lowest AICc); w: Akaike weight.

| **Model selection** | |  |  |  |  |
| --- | --- | --- | --- | --- | --- |
| **Model** | | **df** | **AICc** | **ΔAICc** | **w** |
| rainfall (hatch-band) | | 3 | 107.43 | 0.00 | 0.29 |
| null | | 2 | 108.19 | 0.77 | 0.20 |
| max.mean (hatch-band) | | 3 | 109.79 | 2.36 | 0.09 |
| avg.mean (hatch-band) | | 3 | 110.00 | 2.57 | 0.08 |
| rainfall (fert-hatch) | | 3 | 110.10 | 2.67 | 0.08 |
| max.mean (fert-hatch) | | 3 | 110.22 | 2.79 | 0.07 |
| min.mean (hatch-band) | | 3 | 110.23 | 2.80 | 0.07 |
| min.mean (fert-hatch) | | 3 | 110.32 | 2.89 | 0.07 |
| avg.mean (fert-hatch) | | 3 | 110.33 | 2.90 | 0.07 |
| **Variable** | **Estimate** | | **SE** | **2.5% CI** | **97.5% CI** |
| rainfall (hatch-band) | 0.66 | | 0.42 | -0.16 | 1.48 |
