# Supplementary material for: Influence of winter temperature on nestling sex ratio in the cinereous vulture
Source: PeerJ. 2026 Jun 8;14:e21379. doi: 10.7717/peerj.21379 (PMC13256116; doi:10.7717/peerj.21379)
Supplement: Supplemental Information 3 [file peerj-14-21379-s003.docx]

**Coded categories**

- **Sex**: 0 = female; 1 = male
